# Cardiac fibroblast miR‐27a may function as an endogenous anti‐fibrotic by negatively regulating Early Growth Response Protein 3 (EGR3)

**DOI:** 10.1111/jcmm.15814

**Published:** 2020-11-20

**Authors:** Lifeng Teng, Yubing Huang, Jun Guo, Bin Li, Jin Lin, Lining Ma, Yudai Wang, Cong Ye, Qianqian Chen

**Affiliations:** ^1^ Department of Cardiology Hainan General Hospital Haikou China; ^2^ Department of Cardiology The First Affiliated Hospital of Jinan University GuangZhou China; ^3^ Nursing Department Hainan Maternal and Child Health Hospital Haikou China

**Keywords:** cardiac fibrosis, cardiac remodelling, EGR3, miR‐27a, TGF‐β

## Abstract

Pathological myocardial fibrosis and hypertrophy occur due to chronic cardiac stress. The microRNA‐27a (miR‐27a) regulates collagen production across diverse cell types and organs to inhibit fibrosis and could constitute an important therapeutic avenue. However, its impact on hypertrophy and cardiac remodelling is less well‐known. We employed a transverse aortic constriction (TAC) murine model of left ventricular pressure overload to investigate the in vivo effects of genetic miR‐27a knockout, antisense inhibition of miR‐27a‐5p and fibroblast‐specific miR‐27a knockdown or overexpression. In silico Venn analysis and reporter assays were used to identify miR‐27a‐5p's targeting of Early Growth Response Protein 3 (Egr3). We evaluated the effects of miR‐27a‐5p and Egr3 upon transforming growth factor‐beta (Tgf‐β) signalling and secretome of cardiac fibroblasts in vitro. miR‐27a‐5p attenuated TAC‐induced cardiac fibrosis and myofibroblast activation in vivo, without a discernible effect on cardiac myocytes. Molecularly, miR‐27a‐5p inhibited transforming growth factor‐beta (Tgf‐β) signalling and pro‐fibrotic protein secretion in cardiac fibroblasts in vitro through suppressing the pro‐fibrotic transcription factor Early Growth Response Protein 3 (Egr3). This body of work suggests that cardiac fibroblast miR‐27a may function as an endogenous anti‐fibrotic by negatively regulating Egr3 expression.

## 1 INTRODUCTION

microRNAs (miRNAs) are small non‐coding RNAs (ncRNAs) approximately 22 nucleotides in length that control expression of genes at the level of transcription, by binding to messenger RNAs (mRNAs).^1^ It is becoming increasingly evident that a significant fraction of genes and biochemical pathways are regulated by miRNA or ncRNAs.^2^, ^3^, ^4^ Consequently, and unsurprisingly, miRNAs serve numerous functions under healthy and pathological conditions.^5^ Involvement of miRNAs in regulation of the cardiovascular system is well‐established and includes processes such as chronic stress‐induced cardiac remodelling due to aortic stenosis, which is characterized by fibrosis and hypertrophy.^6^ Among miRNAs known to regulate cardiac fibrosis are miR‐21, miR‐29, miR‐30 and miR‐133, while cardiac hypertrophy is regulated by are miR‐212/132, miR‐133 and miR‐208.^7^, ^8^, ^9^, ^10^, ^11^, ^12^, ^13^, ^14^ Fibrosis and hypertrophy are interrelated and can elicit one another,^15^ and this is borne out by overlapping miRNAs, such as miR‐133, that regulates both these processes.

One notable miRNA—miR‐27a—is dysregulated in both animal models of fibrosis and human fibrotic disease.^16^, ^17^, ^18^, ^19^ Interestingly, miR‐27a has been shown to suppress fibrosis in kidney, bladder, liver and lung pathologies.^16^, ^17^, ^18^, ^19^ These findings suggest that targeting miR‐27a could constitute a possible method to prevent cardiac fibrosis. However, little is known about miR‐27a's role (if any) in cardiac fibrosis. Therefore, better elucidating the net effect of miR‐27a on cardiac remodelling will have important ramifications for its potential as a drug target.

To address this gap in our knowledge, we sought to delineate the involvement of miR‐27a on cardiac remodelling by using both in vivo and in vitro murine models. We demonstrate that genetically or pharmacologically blocking miR‐27a‐5p enhanced myocardial fibrosis in vivo, without a discernible effect on cardiomyocytes (CMs). We also performed in vitro mechanistic studies in cardiac fibroblasts (CFs) to characterize miR‐27a‐5p function more fully and discovered it inhibited the pro‐fibrotic transforming growth factor‐beta (Tgf‐β) signalling pathway through suppressing the pro‐fibrotic transcription factor Early Growth Response Protein 3 (Egr3). This effect resulted in lower CF release of pro‐fibrotic proteins. This body of work highlights the beneficial role of CF miR‐27a‐5p in cardiac remodelling.

## 2 MATERIALS AND METHODS

All experiments received approval from the Ethics Review Committee at Hainan General Hospital (Haikou, China). All mice used in this study were male and were housed and cared for according to the guidelines outlined in the National Institutes of Health's (NIH) ‘Guide for the Care and Use of Laboratory Animals’ (8th edition). The methods are fully detailed in the Supplementary Information.

## 3 RESULTS

### 3.1 Genetic knockout of miR‐27a promotes transverse aortic constriction‐induced heart fibrosis in vivo

**FIGURE 1:**
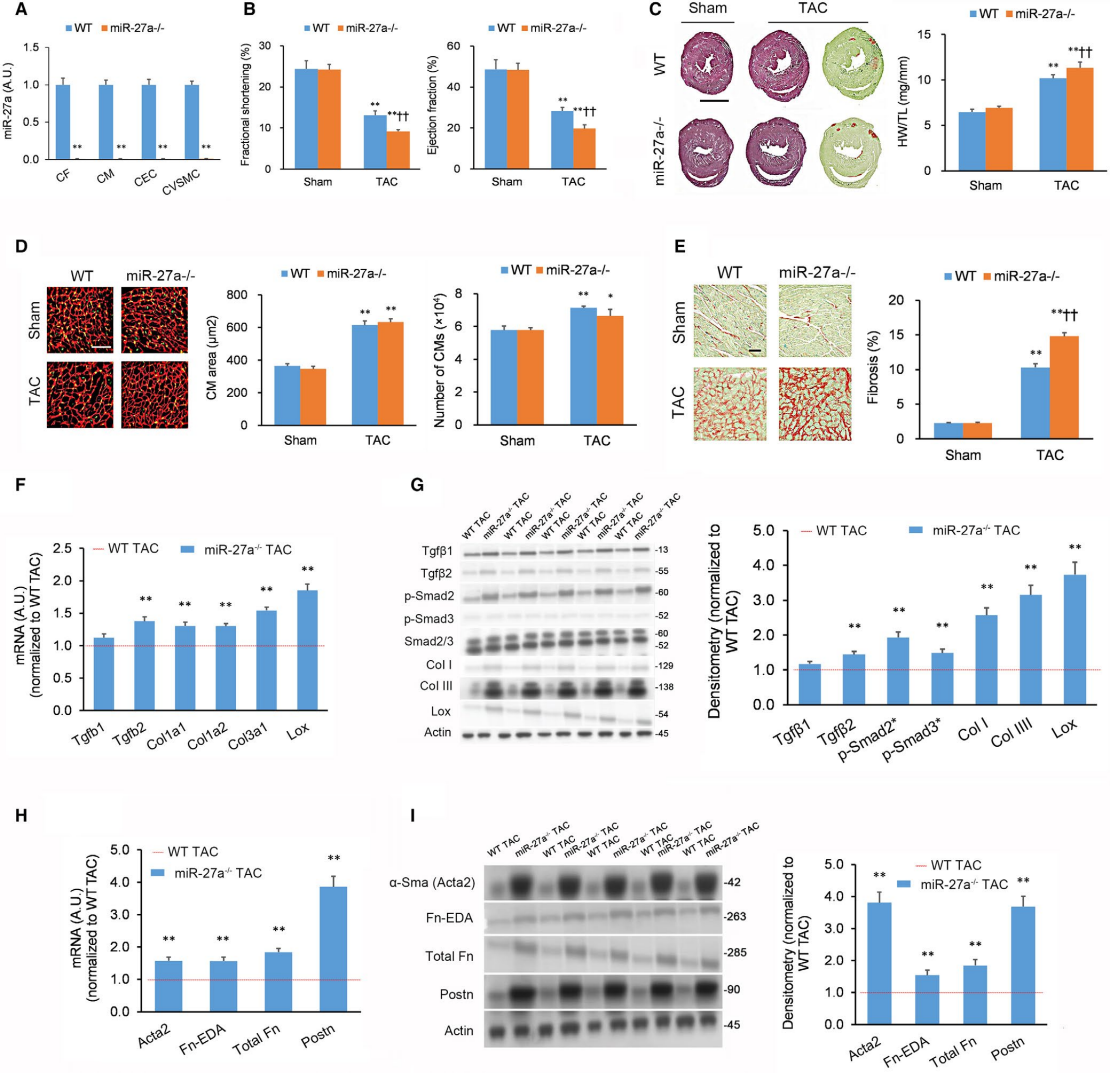
Genetic knockout of miR‐27a promotes transverse aortic constriction‐induced heart fibrosis in vivo. Pulse‐wave Doppler echocardiography and tissue harvesting performed 21 d after transverse aortic constriction (TAC) or sham procedure. n = 9 animals per cohort. (A) miR‐27a levels in cardiac fibroblasts (CFs), cardiomyocytes (CMs), cardiac endothelial cells (CECs) and cardiac vascular smooth muscle cells (CVSMCs) isolated from left ventricular tissue assessed by quantitative real‐time PCR (qPCR) in miR‐27a^−/−^ and wild‐type (WT) animals. (B) Fractional shortening and ejection fraction as measured by echocardiography in TAC‐ or sham‐treated miR‐27a^−/−^ and WT animals. (C) Typical haematoxylin & eosin (H&E) and Picrosirius Red/Fast Green FCF‐stained myocardial sections (scale bar = 2 mm) (left panel); heart hypertrophy evaluated by heart weight‐to‐tibia length ratio (HW/TL) from the cohorts outlined in (B) (right panel). (D) Cardiomyocyte (CM) hypertrophy evaluated in wheat germ agglutinin (WGA)‐stained mid‐ventricular tissue sections from the cohorts outlined in (B); scale bar = 50 µm. CM hypertrophy quantified by CM area and CM count. (E) Extent of fibrosis quantified from Picrosirius Red/Fast Green FCF‐stained cardiac tissue sections from the cohorts outlined in (B). (F, G) Expression of fibrosis markers in left ventricle tissue from TAC‐treated miR‐27a^−/−^ and WT animals quantified by (F) qPCR and (G) Western blotting. (H, I) Expression of myofibroblast activation markers in left ventricle tissue from TAC‐treated miR‐27a^−/−^ and WT animals quantified by (H) qPCR and (I) Western blotting. Full immunoblotting images are displayed in Figure S5. Data expressed as means ± standard errors of the mean (SEMs). For panels (A, F‐I): *P < .05, **P < .01 vs WT or WT TAC [Student's *t* test]. For panels (B‐E): *P < .05, **P < .01 vs matching Sham group; ^†^ *P < *.05, ^††^ *P < *.01 vs WT TAC group [two‐way ANOVA with post hoc Bonferroni]

Since we found that miR‐27a mimics did not affect CM cell size in vitro (Figure S1A,B), we next sought to determine whether manipulation of miR‐27a levels in vivo may affect heart function in models of cardiac disease. For this purpose, we employed mice with genetic knockout (KO) of miR‐27a (miR‐27a^−/−^). As expected, analysing miR‐27a‐5p expression in the four major cardiac cell types revealed miR‐27a‐5p knockdown across the four major cardiac cell types (CFs, CMs, cardiac endothelial cells [CECs] and cardiac vascular smooth muscle cells [CVSMCs]^20^) in miR‐27a^−/−^ mice (Figure 1A). This decrease in miR‐27a‐5p did not affect baseline cardiac characteristics (Figure 1B‐E) or overall body weight (Figure S2A).

Next, we assessed whether diminished miR‐27a‐5p levels would affect cardiac characteristics under pressure overload conditions. Transverse aortic constriction (TAC) procedures were performed to generate a mouse model of pressure overload in the left ventricle. miR‐27a^−/−^ animals that underwent TAC displayed no discernable differences in fibrosis levels in lung, liver and kidney tissues from WT TAC mice (Figure S2B). Notably, miR‐27a^−/−^ TAC mice had inferior left ventricular function than WT TAC mice (Figure 1B). miR‐27a^−/−^ TAC mice exhibited greater levels of heart mass (Figure 1C), left ventricle fibrosis (Figure 1E) and fibrosis markers (*Tgfb2*, *Col1a1, Col1a2, Col3a1*, and *Lox* mRNA levels as well as Tgfβ2, Smad2 phosphorylation, Smad3 phosphorylation, Col I, Col III and Lox protein levels) (Figure 1F,G) compared to WT TAC animals. Moreover, miR‐27a^−/−^ TAC mice exhibited greater levels of myofibroblast activation markers (α‐SMA (Acta2), Fn‐EDA, total Fn and Postn mRNA and protein levels) (Figure 1H,I) compared to WT TAC animals. However, there were no significant effects on CM size, CM counts (Figure 1D) or hypertrophy biomarker expression (*Nppa* transcript expression and *Myh7/Myh6* transcript ratio) (Figure S3A).

### 3.2 Administration of miR‐27a‐5p inhibitor promotes transverse aortic constriction‐induced heart fibrosis in vivo

**FIGURE 2:**
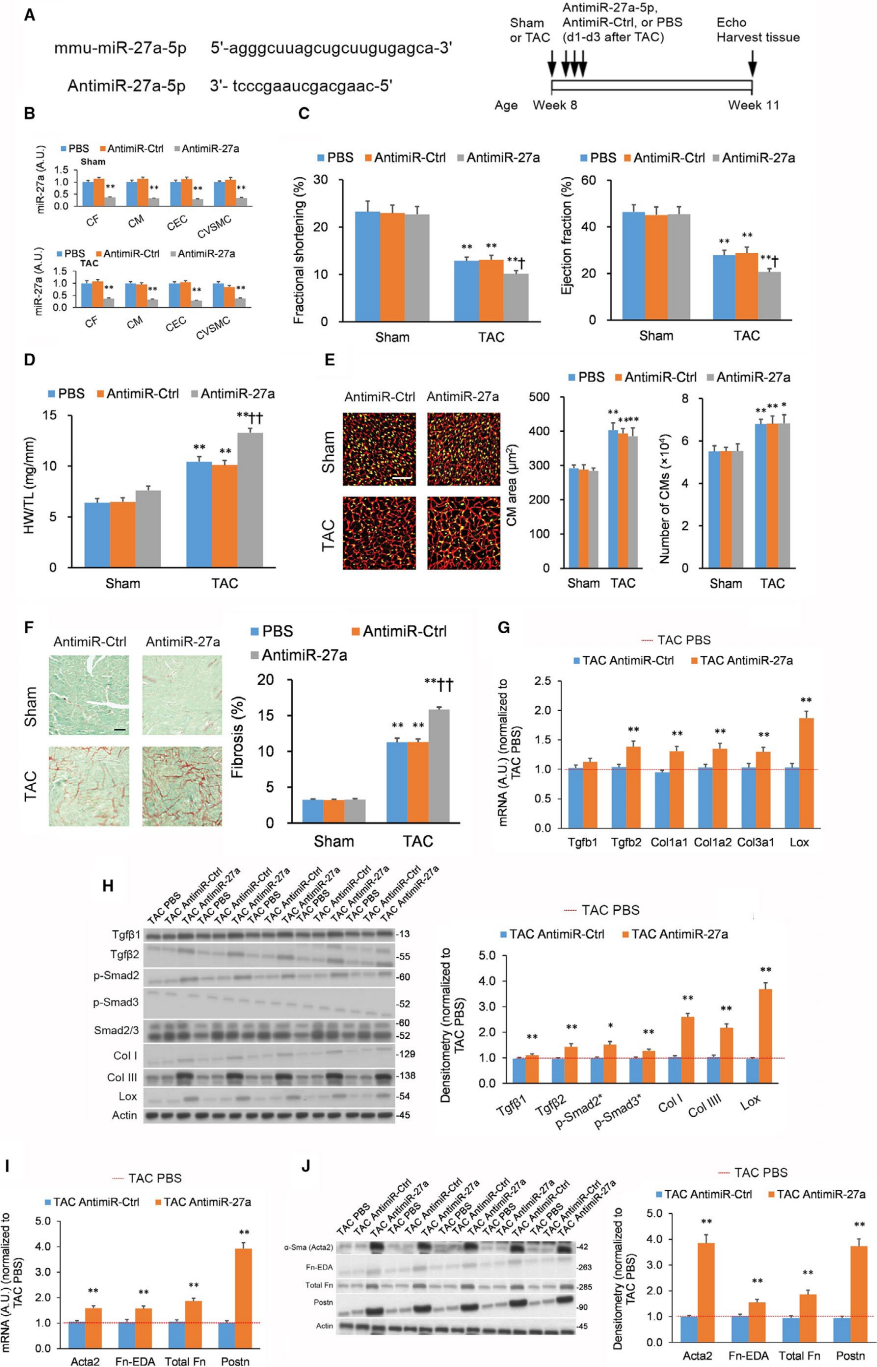
Administration of miR‐27a‐5p inhibitor promotes transverse aortic constriction‐induced heart fibrosis in vivo. Pulse‐wave Doppler echocardiography and tissue harvesting performed 21 d after transverse aortic constriction (TAC) or sham procedure. n = 9 animals per cohort. (A) Design of the locked nucleic acid (LNA) inhibitor against miR‐27a‐5p (left panel) and experimental overview (right panel). (B) miR‐27a‐5p levels in cardiac fibroblasts (CFs), cardiomyocytes (CMs), cardiac endothelial cells (CECs), and cardiac vascular smooth muscle cells (CVSMCs) isolated from left ventricular tissue assessed by quantitative real‐time PCR (qPCR) following anti‐miR‐27a‐5p LNA (Anti‐miR‐27a‐5p), control LNA (Anti‐miR‐Ctrl), or vehicle (PBS) in sham mice (top panel) and TAC mice (bottom panel). (C) Fractional shortening and ejection fraction as measured by echocardiography from the cohorts outlined in (B). (D) Heart hypertrophy evaluated by heart weight‐to‐tibia length ratio (HW/TL) ratio from the cohorts outlined in (B). (E) Cardiomyocyte (CM) hypertrophy evaluated in wheat germ agglutinin (WGA)‐stained mid‐ventricular tissue sections from the cohorts outlined in (B); scale bar = 50 µm. CM hypertrophy quantified by CM area and CM count. (F) Extent of fibrosis quantified from typical Picrosirius Red/Fast Green FCF‐stained left ventricle tissue sections from the cohorts outlined in (B). (G, H) Expression of fibrosis markers in left ventricle tissue from TAC‐treated Anti‐miR‐27a‐5p, Anti‐miR‐Ctrl, and PBS mice quantified by (G) qPCR and (H) Western blotting. (I, J) Expression of myofibroblast activation markers in left ventricle tissue from TAC‐treated Anti‐miR‐27a‐5p, Anti‐miR‐Ctrl and PBS mice quantified by (I) qPCR and (J) Western blotting. Full immunoblotting images are displayed in Figure S5. Data expressed as means ± standard errors of the mean (SEMs). For panels (B, G‐J): *P < .05, **P < .01 vs PBS or TAC PBS [one‐way ANOVA with post hoc Bonferroni]. For panels (C‐F): *P < .05, **P < .01 vs matching Sham group; ^†^ *P < *.05, ^††^ *P < *.01 vs TAC PBS group [two‐way ANOVA with post hoc Bonferroni]

It is possible that compensating mechanisms in miR‐27a^−/−^ animals might conceal other effects. Therefore, we evaluated the effect of acute locked nucleic acid (LNA)‐based miR‐27a‐5p inhibition in adult mice by injecting three doses of an anti‐miR‐27a‐5p LNA that targets mmu‐miR‐27a‐5p (Figure 2A). Anti‐miR‐27a‐5p injections induced a profound decrease in miR‐27a‐5p levels across the four major cardiac cell types (CFs, CMs, CECs and CVSMCs) in comparison to anti‐miR‐Ctrl injections at both baseline and TAC‐induced conditions (Figure 2B). As we had observed in mice with genetic KO of miR‐27a, anti‐miR‐27a‐5p administration reduced left ventricular function in TAC mice (Figure 2C). Anti‐miR‐27a‐5p also promoted heart mass (Figure 2D), left ventricle fibrosis (Figure 2F), fibrosis marker expression (Figure 2G,H) and myofibroblast activation marker expression (Figure 2I,J). Moreover, there were no significant effects on CM size, CM counts (Figure 2E) or hypertrophy biomarker expression (Figure S3B).

### 3.3 Cardiac fibroblast miR‐27a‐5p levels decline with age and transverse aortic constriction‐induced stress

As modulating miR‐27a‐5p expression had an effect on cardiac fibrosis without impacting CM size or counts, we undertook a more in‐depth examination of CF miR‐27a‐5p expression in normal and diseased cardiac tissue. Left ventricle‐derived CF miR‐27a‐5p levels declined with time as mice grew older (Figure S4A), in accordance with its putative role in the regulation of body growth.^21^ Notably, CF miR‐27a‐5p levels dynamically changed following the TAC procedure, with an initial decline 2 days post‐surgery and increases thereafter (Figure S4B). Moreover, miR‐27a‐5p levels in neonatal rat CFs (NRCFs) and adult mouse CFs were much lower after 2 weeks under continuous culture (Figure S4C,D). Cumulatively, these results show that cardiac fibroblast miR‐27a‐5p levels decline with age and TAC‐induced stress, motivating a more in‐depth study on the function of miR‐27a‐5p in CFs.

### 3.4 Selective knockout of cardiac fibroblast miR‐27a‐5p promotes transverse aortic constriction‐induced heart fibrosis in vivo

**FIGURE 3:**
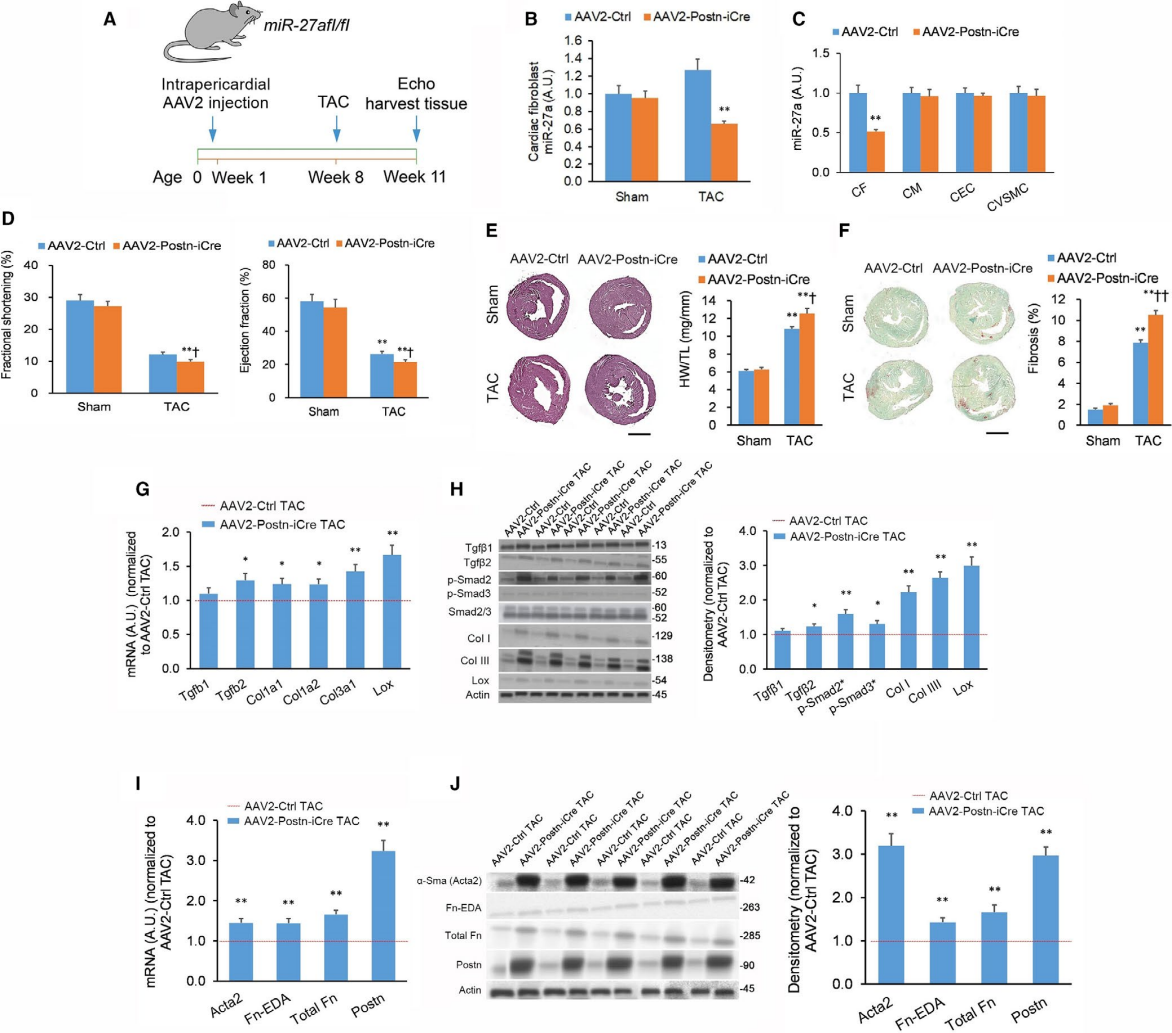
Selective knockout of cardiac fibroblast miR‐27a promotes transverse aortic constriction‐induced heart fibrosis in vivo. (A) miR‐27a^fl/fl^ mice or wild‐type (WT) mice (aged 5 d) were administered tyrosine‐mutant adeno‐associated virus serotype 2 (AAV2^Tyr‐mut^) carrying a copy of enhanced Cre recombinase, or an inactive *C elegans* miR‐39 control sequence, under the control of the murine *Postn* promoter (AAV2^Tyr‐mut^‐Postn‐iCre or AAV2‐Ctrl, 5 × 10^11^ viral particles per µL) by intrapericardial injection. Transverse aortic constriction (TAC) or sham procedure was performed 7 wk post‐ AAV2^Tyr‐mut^ injection. Pulse‐wave Doppler echocardiography and tissue harvesting performed 21 d after TAC or sham procedure. n = 9 animals per cohort. (B) miR‐27a levels in cardiac fibroblasts isolated from left ventricular tissue assessed by quantitative real‐time PCR (qPCR) in TAC‐ or sham‐treated AAV2^Tyr‐mut^‐Postn‐iCre and AAV2^Tyr‐mut^‐Ctrl animals. (C) miR‐27a levels across all major cardiac cell types in left ventricle tissue from TAC‐treated AAV2^Tyr‐mut^‐Postn‐iCre and AAV2^Tyr‐mut^‐Ctrl animals. (D) Fractional shortening and ejection fraction as measured by echocardiography from the cohorts outlined in (B). (E) Typical haematoxylin & eosin (H&E)‐stained heart tissue sections from the cohorts outlined in (B); scale bar = 2 mm. Heart hypertrophy evaluated by heart weight‐to‐tibia length ratio (HW/TL) ratio. (F) Extent of fibrosis quantified from typical Picrosirius Red/Fast Green FCF‐stained cardiac tissue sections from the cohorts outlined in (B); scale bar = 2 mm. (G, H) Expression of fibrosis markers in left ventricle tissue from TAC‐treated AAV2^Tyr‐mut^‐Postn‐iCre and AAV2^Tyr‐mut^‐Ctrl mice quantified by (G) qPCR and (H) Western blotting. (I, J) Expression of myofibroblast activation markers in left ventricle tissue from TAC‐treated AAV2^Tyr‐mut^‐Postn‐iCre and AAV2^Tyr‐mut^‐Ctrl mice quantified by (I) qPCR and (J) Western blotting. Full immunoblotting images are displayed in Figure S5. Data expressed as means ± standard errors of the mean (SEMs). For panels (C, G‐J): *P < .05, **P < .01 vs AAV2^Tyr‐mut^‐Ctrl TAC [Student's *t* test]. For panels (B, D‐F): *P < .05, **P < .01 vs matching Sham group; ^†^ *P < *.05, ^††^ *P < *.01 vs AAV2^Tyr‐mut^‐Ctrl TAC group [two‐way ANOVA with post hoc Bonferroni]

We employed a tyrosine‐mutant adeno‐associated virus serotype 2 vector (AAV2^Tyr‐mut^) under the control of the murine *Postn* promoter (AAV2^Tyr‐mut^‐Postn) that selectively drives gene overexpression in murine CFs.^22^ Selective miR‐27a inactivation in CFs was achieved using AAV2^Tyr‐mut^‐Postn carrying a copy for an enhanced Cre recombinase (AAV2^Tyr‐mut^‐Postn‐iCre) in mice with floxed insertion within the *miR‐27a* allele (miR‐27a^fl/fl^) (Figure 3A). Due to more pronounced *Postn* promoter activity in TAC‐induced activated murine CFs,^22^ miR‐27a^fl/fl^ TAC mice treated with the AAV2^Tyr‐mut^‐Postn‐iCre exhibited lower CF miR‐27a‐5p levels vs WT TAC mice treated with the AAV2^Tyr‐mut^‐Postn‐iCre or mice treated with the AAV2‐Ctrl (Figure 3B); this miR‐27a‐5p knockdown was specific to CFs and did not impact the other cardiac cell types (Figure 3C). miR‐27a^fl/fl^ mice treated with the AAV2^Tyr‐mut^‐Postn‐iCre exhibited reduced left ventricular function (Figure 3D). miR‐27a^fl/fl^ mice treated with the AAV2^Tyr‐mut^‐Postn‐iCre displayed increased cardiac mass (Figure 3E), left ventricle fibrosis (Figure 3F), fibrosis marker expression (Figure 3G,H) and myofibroblast activation marker expression (Figure 3I,J). Moreover, there were no significant effects on hypertrophy biomarker expression (Figure S3C). Overall, selective miR‐27a KO from CFs produced the same phenotypic effect as systemic miR‐27a KO and LNA inhibition of miR‐27a‐5p.

### 3.5 Selective overexpression of cardiac fibroblast miR‐27a‐5p suppresses transverse aortic constriction‐induced heart fibrosis in vivo

**FIGURE 4:**
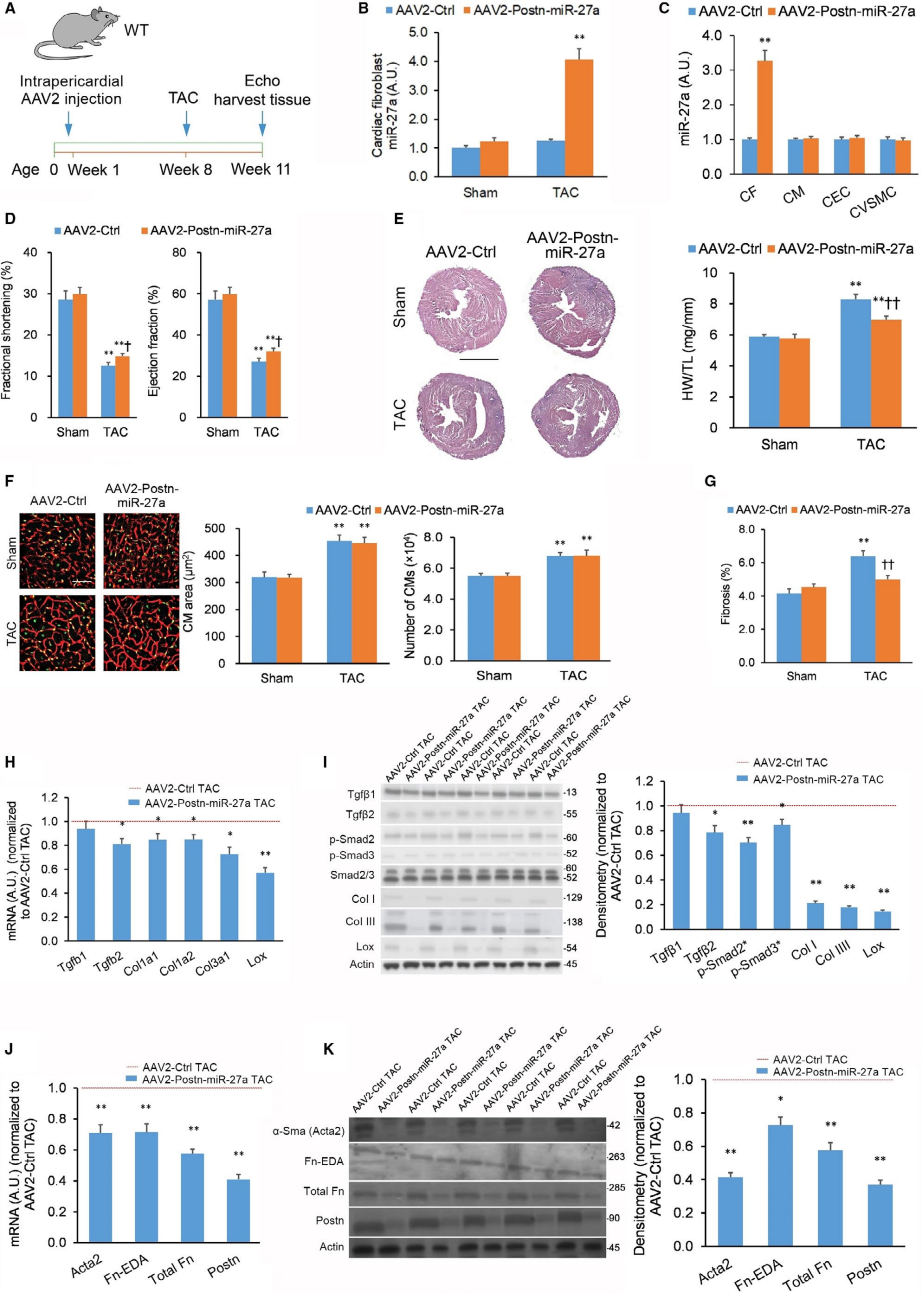
Selective overexpression of cardiac fibroblast miR‐27a suppresses transverse aortic constriction‐induced heart fibrosis in vivo. (A) Wild‐type (WT) animals (aged 5 wk) were administered tyrosine‐mutant adeno‐associated virus serotype 2 (AAV2^Tyr‐mut^) carrying a copy of miR‐27a, or an inactive *C elegans* miR‐39 control sequence, under the control of the murine *Postn* promoter (AAV2^Tyr‐mut^‐Postn‐iCre or AAV2^Tyr‐mut^‐Ctrl, 5 × 10^11^ viral particles per µL) by intrapericardial injection. Transverse aortic constriction (TAC) or sham procedure was performed 7 wk post‐ AAV2^Tyr‐mut^ injection. Pulse‐wave Doppler echocardiography and tissue harvesting performed 21 d after TAC or sham procedure. n = 9 animals per cohort. (B) Cardiac fibroblast miR‐27a levels in left ventricle tissue assessed by quantitative real‐time PCR (qPCR) in TAC‐ or sham‐treated AAV2^Tyr‐mut^‐Postn‐miR‐27a and AAV2^Tyr‐mut^‐Ctrl animals. (C) miR‐27a levels across all major cardiac cell types in left ventricle tissue from TAC‐treated AAV2^Tyr‐mut^‐Postn‐miR‐27a and AAV2^Tyr‐mut^‐Ctrl animals. (D) Fractional shortening and ejection fraction as measured by echocardiography from the cohorts outlined in (B). (E) Typical haematoxylin & eosin (H&E)‐stained heart tissue sections from the cohorts outlined in (B); scale bar = 2 mm. Heart hypertrophy evaluated by heart weight‐to‐tibia length ratio (HW/TL) ratio. (F) Cardiomyocyte (CM) hypertrophy evaluated in wheat germ agglutinin (WGA)‐stained mid‐ventricular tissue sections from the cohorts outlined in (B); scale bar = 50 µm. CM hypertrophy quantified by CM area and CM count. (G) Assessment of fibrosis in left ventricle cardiac tissue sections from the cohorts outlined in (B). (H, I) Expression of fibrosis markers in left ventricle tissue from TAC‐treated AAV2^Tyr‐mut^‐Postn‐miR‐27a and AAV2^Tyr‐mut^‐Ctrl mice quantified by (H) qPCR and (I) Western blotting. (J, K) Expression of myofibroblast activation markers in left ventricle tissue from TAC‐treated AAV2^Tyr‐mut^‐Postn‐miR‐27a and AAV2^Tyr‐mut^‐Ctrl mice quantified by (J) qPCR and (K) Western blotting. Full immunoblotting images are displayed in Figure S5. Data expressed as means ± standard errors of the mean (SEMs). For panels (C, H‐K): *P < .05, **P < .01 vs AAV2^Tyr‐mut^‐Ctrl TAC [Student's *t* test]. For panels (B, D‐G): *P < .05, **P < .01 vs matching Sham group; ^†^ *P < *.05, ^††^ *P < *.01 vs AAV2^Tyr‐mut^‐Ctrl TAC group [two‐way ANOVA with post hoc Bonferroni]

A reverse approach was also performed in which miR‐27a‐5p was selectively overexpressed (OE) in CFs in vivo using the same tyrosine‐mutant AAV2^Tyr‐mut^ vector under the control of the *Postn* promoter (AAV2^Tyr‐mut^‐Postn‐miR‐27a) (Figure 4A). Due to more pronounced *Postn* promoter activity in TAC‐induced activated murine CFs,^22^ WT TAC mice receiving AAV2^Tyr‐mut^‐Postn‐miR‐27a expressed higher CF miR‐27a‐5p levels vs WT TAC mice treated with AAV2^Tyr‐mut^‐Postn‐Ctrl or mice treated with the AAV2^Tyr‐mut^‐Ctrl (Figure 4B); this miR‐27a‐5p knockdown was specific to CFs and did not impact other cardiac cell types (Figure 4C). Upon undergoing a TAC procedure, AAV2^Tyr‐mut^‐Postn‐miR‐27a mice exhibited reduced left ventricular function (Figure 4D). Moreover, AAV2^Tyr‐mut^‐Postn‐miR‐27a TAC mice exhibited lower heart mass with no effect on CM size or counts (Figure 4E,F). AAV2^Tyr‐mut^‐Postn‐miR‐27a TAC mice exhibited reduced left ventricular fibrosis (Figure 4G), lower fibrosis marker expression (Figure 4H,I), and lower myofibroblast activation marker expression (Figure 4J,K). Moreover, there were no significant effects on hypertrophy biomarker expression (Figure S3D). The results from selective miR‐27a‐5p OE in CFs in vivo support the beneficial function of CF miR‐27a‐5p in cardiac remodelling.

### 3.6 miR‐27a‐5p suppresses cardiac fibroblast's pro‐fibrotic activity via Early Growth Response Protein 3 (Egr3) in vitro

**FIGURE 5:**
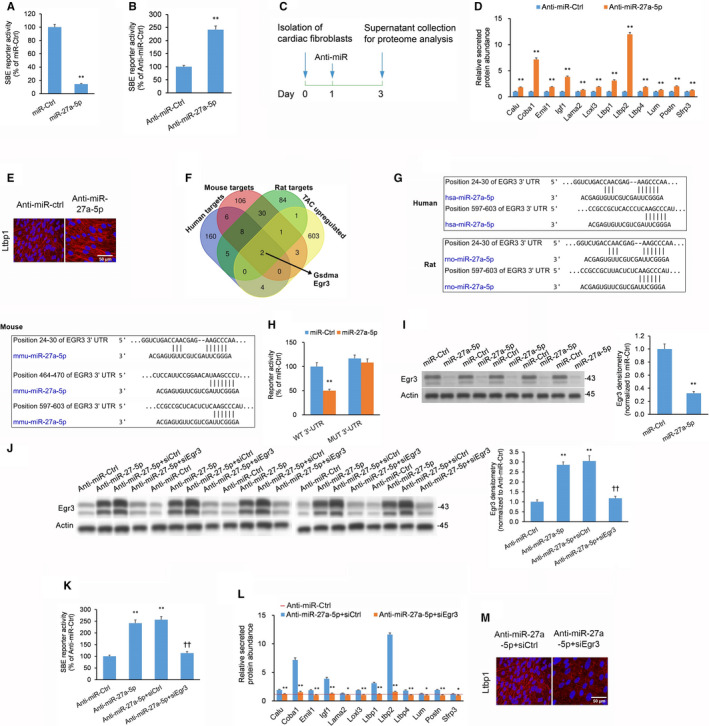
miR‐27a‐5p suppresses pro‐fibrotic activity in cardiac fibroblasts via Egr3 in vitro. (A, B) Transforming growth factor‐beta (Tgf‐β) activity in NRCFs assessed by SBE luciferase reporter assays 48 h after treatment with (A) miR‐27a‐5p mimic or miR‐Ctrl or (B) locked nucleic acid (LNA) against miR‐27a‐5p (Anti‐miR‐27a‐5p) or control LNA (Anti‐miR‐Ctrl). (C) Experimental overview for neonatal rat cardiac fibroblast (NRCF) secretome analysis. (D) Differential secretion of pro‐fibrotic proteins by NRCFs treated with Anti‐miR‐27a‐5p expressed by fold‐change relative to NRCFs treated with Anti‐miR‐Ctrl. (E) Immunofluorescent imaging showing extracellular secretion of Ltbp1 by NRCFs treated with Anti‐miR‐27a or Anti‐miR‐Ctrl. Nuclei stained blue with DAPI. (F) In silico Venn analysis of putative hsa‐miR‐27a‐5p (human) targets (blue), mmu‐miR‐27a‐5p (mouse) targets (red), rno‐miR‐27a‐5p (rat) targets (green), and up‐regulated genes in the TAC murine left ventricular transcriptome (yellow). (G) Targetscan analysis identifies two conserved miR‐27a‐5p binding sites in Egr3's 3′ untranslated region (3′‐UTR) across humans, mice, and rats. (H) Dual‐fluorophore reporter assay of miR‐27a‐5p binding to wild‐type (WT) or mutated (MUT) EGR3 3′‐UTR in HEK293 cultures that underwent co‐transfection with miR‐27a‐5p mimic or miR‐Ctrl; ratiometric calculations performed with first fluorophore eGFP (miR‐27a‐5p binding standard) and second fluorophore tdTomato (transfection standard). (L) Differential secretion of pro‐fibrotic proteins by NRCFs treated with Anti‐miR‐27a‐5p + siEgr3 and Anti‐miR‐27a‐5p + siCtrl expressed by fold‐change relative to NRCFs treated with Anti‐miR‐Ctrl. (M) Immunofluorescence showing extracellular secretion of Ltbp1 by NRCFs treated with Anti‐miR‐27a‐5p + siEgr3 or Anti‐miR‐27a‐5p + siCtrl. Nuclei stained blue with DAPI. Full immunoblotting images are displayed in Figure S5. All experiments: n = 3 biological replicates × 3 technical replicates. Data expressed as means ± standard errors of the mean (SEMs). For panels (A, B, D, I): *P < .05, **P < .01 vs Anti‐miR‐Ctrl or miR‐Ctrl group [Student's *t* test]. For panel (H): *P < .05, **P < .01 vs matching miR‐Ctrl group [two‐way ANOVA with post hoc Bonferroni]. For panel (J, K): *P < .05, **P < .01 vs Anti‐miR‐Ctrl group; †*P < *.05, ††*P < *.01 vs Anti‐miR‐27a‐5p group [one‐way ANOVA with post hoc Bonferroni]. For panel (L): *P < .05, **P < .01 vs Anti‐miR‐27a‐5p + siCtrl [Student's *t* test]

Our in vivo experiments showed that miR‐27a‐5p displays an anti‐fibrotic effect in TAC mice. We have been suggested that miR‐27a‐5p may modulate Tgf‐β signalling activity in CFs. Indeed, miR‐27a‐5p mimic in NRCFs decreased bioluminescence in a SBE reporter assay of Tgf‐β activity (Figure 5A), while anti‐miR‐27a‐5p LNA produced the opposite effect (Figure 5B). We also evaluated the secretome of NRCFs to determine whether miR‐27a‐5p was associated with secretion of fibrosis‐related proteins from NRCFs. Following transfection of NRCFs with anti‐miR‐27a‐5p LNA, the conditioned media were harvested and subjected to proteomic analysis by tryptic digest followed by mass spectrometry (Figure 5C). Anti‐miR‐27a‐5p LNA in NRCFs increased the secretion of multiple mediators of fibrosis compared to NRCFs transfected with anti‐miR‐Ctrl LNA (Figure 5D). Many of the genes encoding for the secreted factors have been associated with the Tgf‐β signalling cascade, such as lysyl oxidase‐like 3 (Loxl3)^23^ and the latent Tgf‐β binding proteins (Ltbp1, Ltbp2).^24^ Using immunofluorescence, we confirmed that anti‐miR‐27a‐5p LNA enhanced Ltbp1 secretion from NRCFs (Figure 5E). These combined observations imply that miR‐27a‐5p negatively regulates pro‐fibrotic activity in cardiac fibroblasts.

We then conducted an in silico Venn analysis to determine the target gene(s) of miR‐27a‐5p that may be responsible for miR‐27a‐5p's anti‐fibrotic action in CFs. We analysed the overlapping putative TargetScan‐derived target genes for human, mouse and rat miR‐27a‐5p that were also up‐regulated in the left ventricular transcriptome of WT TAC mice relative to WT sham mice (GEO accession: GSE18224 ^25^). This Venn analysis uncovered gasdermin (Gsdma) and Egr3 as potential targets of miR‐27a‐5p that are up‐regulated by TAC (Figure 5F). Considering that Gsdma is an apoptosis mediator primarily expressed in epithelial cells and T‐lymphocytes^26^ while Egr3 is a transcription factor involved in the Tgf‐β‐induced fibrotic response in fibroblasts,^27^ we selected Egr3 for further analysis. Follow‐up TargetScan analysis revealed two putative miR‐27a‐5p binding sites in the 3′‐untranslated region (3′‐UTR) of Egr3 that are conserved across human, mice and rats (Figure 5G). Through mutating these two conserved miR‐27a‐5p binding sites in the EGR3 3′‐UTR, our 3′‐UTR reporter assay in HEK293 cells revealed that the WT EGR3 3′‐UTR is a direct regulatory target of miR‐27a‐5p (Figure 5H). We confirmed that miR‐27a‐5p mimic in NRCFs decreased Egr3 protein expression (Figure 5I). We also showed that anti‐miR‐27a‐5p LNA in NRCFs increased Egr3 protein expression, an effect abrogated by addition of a small‐interfering RNAs against Egr3 (siEgr3) (Figure 5J). Moreover, anti‐miR‐27a‐5p LNA in NRCFs increased SBE reporter bioluminescence, an effect abrogated by addition of siEgr3 (Figure 5K).

Next, we determined whether Egr3 silencing could block anti‐miR‐27a‐5p LNA‐elicited secretion of pro‐fibrotic factors by NRCFs. siEgr3 blocked anti‐miR‐27a‐5p LNA‐triggered secretion of pro‐fibrotic mediators (Figure 5L). By immunofluorescence, we confirmed that the addition of siEgr3 reduced Ltbp1 deposition from anti‐miR‐27a‐5p LNA‐treated NRCFs (Figure 5M). Overall, our findings advocate that miR‐27a negatively regulates pro‐fibrotic Tgf‐β activity in CFs via Egr3.

## 4 DISCUSSION

Herein, we performed a series of experiments that cumulatively support miR‐27a‐5p's suppression of pathological cardiac fibrosis during heart remodelling. Selective genetic KO of miR‐27a or miR‐27a‐5p LNA‐based inhibition enhanced cardiac stress‐induced fibrosis in mice that had undergone TAC procedure without impacting CMs, whereas selective OE of CF miR‐27a‐5p produced the reverse outcome. Secretome analysis in CFs identified several pro‐fibrotic factors that were differentially expressed upon miR‐27a KD. Luminescence reporter assays of Egr3 3′‐UTR binding pointed to the pro‐fibrotic transcription factor Egr3 as a major mediator of miR‐27a‐5p's suppressive effect on fibrosis.

Several pathological illnesses stimulate fibrosis and the release of extracellular matrix (ECM) proteins. miR‐27a has been shown to suppress fibrosis under pathological conditions in the kidney, bladder, and liver *in vivo* ^16^, ^17^, ^18^ and CF collagen gene expression in vitro.^12^ Therefore, here we have been suggested that miR‐27a‐5p may have an anti‐fibrotic effect in the heart. The prominent anti‐fibrotic effect of miR‐27a‐5p in CFs is indicated by several lines of evidence: (a) CF miR‐27a‐5p levels decline with age and TAC‐induced stress; (b) selective KO of miR‐27a from CFs using CF‐targeting AAV2^Tyr‐mut^‐Postn‐iCre in miR‐27a^fl/fl^ mice worsens TAC‐elicited cardiac fibrosis, while selective miR‐27a‐5p OE in WT mice CFs improves TAC‐triggered pathology; and (c) TAC‐triggered increases in fibrosis marker and myofibroblast activation marker expression are heightened in mice with selective CF miR‐27a KO and lowered in mice with selective CF miR‐27a OE.

Our 3′‐UTR reporter and immunoblotting assays identified Egr3 as a direct regulatory target of miR‐27a‐5p. We also found that miR‐27a‐5p KD in NRCFs in vitro promotes pro‐fibrotic factor secretion in an Egr3‐dependent manner. Egr3 is a member of the Early Growth Response (Egr) family of transcription factors (Egr‐1, Egr‐2 and Egr‐4) that all share a conserved zinc‐finger domain targeting the Egr response element present in several gene promoters.^28^ Specifically, Egr3 has been shown to be a TGF‐ß‐induced transcription factor that bolsters pro‐fibrotic gene expression in human fibroblasts.^27^ Moreover, murine fibroblasts with Egr3 knockout show down‐regulated levels of several key fibrotic genes (ie Col1a1, Acta2, Tgfß1, Ctgf and Pai1) in response to Tgf‐ß2 stimulus, revealing that Egr3 is necessary and sufficient for Tgf‐β‐induced fibrotic responses.^27^ This is notable considering the Tgf‐β2 up‐regulation observed in our TAC mouse left ventricular tissue samples. Our proposition is that miR‐27a‐5p suppresses Tgf‐β‐induced cardiac fibrosis by inhibiting Egr3 expression in CFs.

Additional research could shed light on several aspects not yet investigated. First, mice can be followed for longer durations after TAC surgery to see if, and to what extent, miR‐27a‐5p OE decreases cardiac fibrosis over time. Second, researchers can further elucidate the molecular details of miR‐27a‐5p's mechanism of action to determine how the miR‐27a‐5p/Egr3 axis affects CF‐mediated myocardial fibrosis and the significance of the Tgf‐β cascade in this biological process. Third, it remains unknown whether miR‐27a‐5p adopts different distributions in other organs, and whether it shares any overlap in anti‐fibrotic function in these organs. We partially addressed this question here, as the impact of miR‐27a‐5p KO on organ fibrosis was examined in lung, liver and kidney tissues in miR‐27a^−/−^ animals following TAC. Histological analysis of these organs did not reflect any ascertainable changes in fibrosis. However, miR‐27a‐5p's role in fibrotic disease models of these organs remains to be elucidated.

Cumulatively, our results underscore the beneficial role of miR‐27a‐5p in cardiac remodelling. Other animal models, such as the left‐anterior descending coronary artery myocardial infarction (LAD MI) model and Ang infusion model, are needed to confirm the therapeutic potential of miR‐27a‐5p agomiR therapy in vivo.

## CONFLICT OF INTEREST

The authors confirm that there are no conflicts of interest.

## AUTHOR CONTRIBUTIONS

**Lifeng Teng:** Conceptualization (equal); data curation (equal). **Yubing Huang:** formal analysis (equal); investigation (equal). **Jun Guo:** Data curation (equal); formal analysis (equal); software (equal); writing – original draft (equal). **Bin Li:** Data curation (equal); formal analysis (equal); software (equal). **Jin Lin:** Data curation (equal); formal analysis (equal); software (equal). **Lining Ma:** Data curation (equal); resources (equal); validation (equal). **Yudai Wang:** Data curation (equal); resources (equal); validation (equal). **Cong Ye:** Formal analysis (equal); validation (equal); visualization (equal). **Qianqian Chen:** Data curation (equal); resources (equal); validation (equal).

## Supporting information

Supplementary Material

## Data Availability

The data that support the findings of this study are available on request from the corresponding author. The data are not publicly available due to privacy or ethical restrictions.
