# Supplementary material for: Cardiac fibroblast miR‐27a may function as an endogenous anti‐fibrotic by negatively regulating Early Growth Response Protein 3 (EGR3)
Source: J Cell Mol Med. 2020 Nov 20;25(1):73–83. doi: 10.1111/jcmm.15814 (PMC7810947; doi:10.1111/jcmm.15814)
Supplement: Supplementary file 1 — Supplementary Material [file JCMM-25-73-s001.docx]

**SUPPLEMENTARY INFORMATION FOR**

**Cardiac fibroblast miR-27a may function as an endogenous anti-fibrotic by negatively regulating Early Growth Response Protein 3 (EGR3)**

**SUPPLEMENTARY METHODS**

**Procurement of conventional miR-27a knockout mice and miR-27a floxed mice**

Conventional miR-27a knockout (KO) (miR-27a^−/−^) mice on a C57BL/6N background were obtained from the Tongji University School of Medicine (Shanghai, China) and had been produced according to an established CRISPR/Cas9 procedure [[1](#_ENREF_1)]. Briefly, *in vitro*-translated Cas9 mRNA and gRNA were co-microinjected into C57BL/6N murine zygotes. The three gRNA sequences used to generate the miR-27a KO mice were as follows: GAG AAG CCT ATC ATG ACA AC, CTG TGA ACA CGA CTT TGC TG, and AGT GGC TAA GTT CCG CCC CC. Founders with frameshift mutations were screened via T7E1 assay and validated through DNA sequencing. One of F1 miR-27a^−/−^ founder mice was chosen to backcross to C57BL/6N wild-type (WT) mice for more than five generations to maintain the KO strain. Our lab employed routine PCR to validate the miR-27a^−/−^ genotype.

miR-27a floxed (miR-27a^fl/fl^) mice on a C57BL/6N background were also obtained from the Tongji University School of Medicine (Shanghai, China) and had been produced according to a conventional procedure [[2](#_ENREF_2)]. Briefly, a miR-27a targeting vector was designed to insert LoxP sites on either side of the miR-27a coding region in C57BL/6N murine embryonic stem (ES) cells using standard protocols. A neomycin resistance cassette was used for positive selection, while a TK cassette was used for negative selection. Appropriately-targeted clones were identified using Southern blotting of digested gDNA with a probe external to the miR-27a targeting vector. Appropriately-targeted ES cells were then injected into C57BL/6N blastocysts to produce C57BL/6N chimeras. Germline-transmitting chimeras were crossed to C57BL/6N Flpe transgenic mice in order to remove the neomycin cassette, thereby producing miR-27a^fl/fl^ mice. Our lab employed routine PCR to validate the miR-27a^fl/fl^ genotype.

**Generation of AAV2 viral vectors**

A triple tyrosine-mutant adeno-associated virus serotype 2 (AAV2^Tyr-mut^) vector (with Y444F, Y500F, and Y730F mutations) targeting fibroblasts [[3](#_ENREF_3)] was placed under the control of the 3.9-kb murine periostin (*Postn*) promoter to create the AAV2^Tyr-mut^-Postn vector that selectively drives gene overexpression in murine cardiac fibroblasts (CFs) [[4](#_ENREF_4)]. A 700-bp fragment that encompassed the miR-27a-5p precursor sequence was amplified from murine gDNA and ligated into AAV2^Tyr-mut^-Postn to create AAV2^Tyr-mut^-Postn-miR-27a-5p. As a control, AAV2^Tyr-mut^-Postn-Ctrl was created from a 350-bp sequence encoding *C. elegans* miR-39, which was ligated into AAV2^Tyr-mut^-Postn. A codon-optimized Cre-recombinase (iCre) was ligated into AAV2-Postn to generate AAV2^Tyr-mut^-Postn-iCre.

HEK293 cells in triple flasks in Dulbecco's Modified Eagle Medium (DMEM) with 10% fetal bovine serum (FBS) underwent transfection with polyethylenimine (Sigma-Aldrich) of: (i) AAV2^Tyr-mut^ vectors (either AAV2^Tyr-mut^-Postn-miR-27a-5p, AAV2^Tyr-mut^-Postn-Ctrl, or AAV2^Tyr-mut^-Postn-iCre), and (ii) the pDP9rs helper plasmid (generous donation from the Icahn School of Medicine at Mount Sinai). Benzonase was added to the HEK293 cells, which were lysed, and viral isolation was by OptiPrep Density Gradient Medium (Sigma-Aldrich). Quantitative real-time PCR (qPCR) was used to quantify AAV2^Tyr-mut^ titers with SYBR Green Master Mix (Roche).

**Experimental modulation of miR-27a expression**

For the locked nucleic acid (LNA) experiments, antimiR-27a-5p LNA (3’-TCC CGA AUC GAC GAAC-5’) or antimiR-Ctrl LNA (negative control A sequence) (both miRCURY LNA miRNA Power Inhibitors, Qiagen) was administered (i.v.) at a dose of 20 mg/kg in PBS in three separate injections given over three consecutive days (days 1-3 post-operation) as previously described [[5](#_ENREF_5)]. An equivalent volume of PBS was injected in an identical manner as a negative control.

For CF-selective knockout of miR-27a-5p, miR-27a^fl/fl^ C57BL/6N pups (aged 3-4 days) received AAV2^Tyr-mut^-Postn-iCre by intrapericardial injection according to established procedure [[6](#_ENREF_6)]. In brief, miR-27a^fl/fl^ pups underwent anesthesia with medetomidine (0.5 mg/kg), midazolam (5 mg/kg), and fentanyl (0.05 mg/ kg) administration, followed by intrapericardial injection of 50 µl of AAV2^Tyr-mut^-Postn-iCre or AAV2^Tyr-mut^-Ctrl (1.0 × 10^10^ viral particles per µl) using a 30 G needle.

For CF-selective overexpression of miR-27a-5p, WT C57BL/6N pups (aged 3-4 days) underwent anesthesia as described above and received intrapericardial injection of 50 µl of either AAV2^Tyr-mut^-Postn-miR-27a-5p or AAV2^Tyr-mut^-Ctrl (1.0 × 10^10^ viral particles per µl) using a 30 G needle.

**Transverse aortic constriction mouse model**

C57BL/6N mice (aged 8 weeks old; Charles River Laboratories) underwent transverse aortic constriction (TAC) according to an established procedure with some modifications [[7](#_ENREF_7)]. Animals were first administered buprenorphine (0.1 mg/kg, s.c.) and intubated with isoflurane an hour later. A thoracic incision was made between the 2^nd^ and 3^rd^ ribs and the aortic arch was ligated with a cannula (27 G). Sham procedures consisted of opening the thoracic cavity without performing an aortic ligation. All animals were supervised for 2-4 hours while they recovered under a heating lamp. Pulse-wave Doppler echocardiograms assessed heart size and function before sham/TAC procedure and before the experimental endpoint when animals were euthanized.

For euthanization, a polyethylene catheter with heparinized (100 IU/ml) phosphate buffer (0.2 M, pH 7.4) was inserted through the right carotid artery into the ascending aorta. The heart was arrested in diastole by injecting 1-ml mixture of cadmium chloride (100 mM) and potassium chloride (3 M) in rapid succession through an aortic catheter as previously described [[8](#_ENREF_8)]. The whole heart was excised and perfused in a retrograde manner with non-heparinized phosphate buffer for three minutes to flush out residual blood from the coronary tree. Heart weights were measured prior to freezing down at -80°C.

**Histological and immunohistochemistry (IHC) analysis**

Histological and IHC analysis was performed on frozen or fixed mouse ventricular tissue samples where appropriate. Ventricular tissue was fixed in PFA (4%), paraffin-embedded, sectioned, and stained by hematoxylin & eosin (H&E). Mouse ventricular tissue sections (8 µm-thickness) were stained by Picrosirius Red and Fast Green FCF to quantify collagen deposits, expressed as the percent of total area that stained red (indicative of collagen). Transverse tissue sections (6 µm-thickness) were stained with Alexa Fluor 647-tagged wheat germ agglutinin (WGA, 1:200; Life Technologies, Thermo Fisher Scientific) to delineate cardiomyocyte (CM) contours and with SYTOX Green Nucleic Acid Stain (1:1000; Life Technologies) to delineate nuclei. CM cross-sectional area was quantified in images that were captured using a Leica TCS SP5 II confocal microscope (Leica Microsystems) fitted with a 20× objective, and 488 nm (SYTOX Green) and 633 nm (WGA) lasers. Cells were automatically identified in the WGA channel by MetaMorph Microscopy Automation and Image Analysis Software (Molecular Devices) using thresholds fixed to exclude background and excessive fibrosis. Mean CM area was calculated with the morphometry module in MetaMorph. CM counts were made from tissue sections stained with WGA and anti-cTNT (Thermo Fisher Scientific) to identify CMs and imaged by fluorescence microscopy. A blinded investigator utilized ImageJ (<https://imagej.nih.gov/ij/>) to manually count CMs within a 0.1 mm^2^ area, and total CM counts were projected by scaling to total area.

**Isolation of murine cardiomyocytes, cardiac fibroblasts, endothelial cells, and vascular smooth muscle cells**

Adult mouse cardiomyocytes (CMs), CFs, cardiac endothelial cells (CECs), and cardiac vascular smooth muscle cells (CVSMCs) were isolated from adult C57BL/6N mouse left ventricular tissue that had undergone sham or TAC. For the experiments directly referencing isolated CMs, CFs, CECs, and CVSMCs, left ventricular tissue was fragmented, digested, and subjected to cell isolation procedures as previously described with minor modifications [[9](#_ENREF_9)].

For CEC isolation, tissue fragments were digested in PBS supplemented with 2 mg/ml collagenase type II (Worthington Biochemical Corporation) for 60 minutes (37°C, 5% CO_2_). After 70-µm filtration and red blood cell lysis (Miltenyi Biotec), cells were washed and resuspended in MACS buffer (Miltenyi Biotec). After 30-µm filtration, CECs were isolated with the mouse CD31 MicroBead Kit using the MACS system (Miltenyi Biotec). The resulting CD31+ eluate (containing the purified CECs) were cultured in Endothelial Cell Growth Medium (Sigma-Aldrich) until 80-90% confluence prior to analysis.

For CM, CF, and CVSMC isolation, tissue fragments were digested in buffer A (10 mM HEPES, 0.6 mM KH_2_PO_4_, 0.6 mM Na_2_HPO_4_, 12 mM NaHCO_3_, 10 mM KHCO_3_, 113 mM NaCl, 4.7 mM KCl, 1.2 mM MgSO_4_, and 30 mM taurine) supplemented with 2 mg/ml collagenase type II for 2.5 hours (37°C, 5% CO_2_). The digested tissue was dissociated with a syringe for one minute and transferred to buffer B [47.5 ml buffer A, 2.5 ml fetal calf serum (FCS; Sigma-Aldrich), and 62.5 ml CaCl_2_ (10 μM)] and incubated for another 5 minutes with the CaCl_2_ concentration gradually raised to 100 µM for calcium reconstitution. The mixed cell suspension underwent 100-µm filtration and was transferred to minimal essential medium (MEM) supplemented with 5% FCS, 2 mM L-glutamine, 10 mM 2,3-butanedione monoxime, and 1% penicillin/streptomycin (pen-strep).

CMs, CFs, and CVSMCs in the mixed cell suspension were then separated from each other as previously described with minor modifications [[10](#_ENREF_10)]. In brief, CMs were gravity-sedimented for 10 minutes (4°C). The remaining supernatant (containing the suspended CFs and CVSMCs) was subjected to 40-μm filtration, washed, and resuspended in MACS buffer (Miltenyi Biotec). The sedimented CMs were resuspended and cultured in MEM supplemented with 5% FCS and 1% pen-strep until 60-70% confluence prior to analysis. CF purification was performed with the mouse/rat CD90.1 MicroBead Kit using the MACS system (Miltenyi Biotec). The resulting CD90+ eluate (containing the purified CFs) was resuspended and cultured in MEM supplemented with 5% FCS and 1% pen-strep until 80-90% confluence prior to analysis. The remaining effluent (primarily containing CVSMCs and CECs) was filtered for CECs using the mouse CD31 MicroBead Kit as described above, and the remaining effluent (containing the purified CVSMCs) was resuspended in RPMI 1640 medium with 5% FCS and 1% pen-strep until 80-90% confluence prior to analysis. Plated CVSMCs were confirmed by smooth muscle α-actin and smooth muscle myosin heavy chain staining.

**Flow cytometric validation of murine heart-derived fibroblast cell fraction purity**

The purity of the murine heart-derived CF cell fraction was characterized via fluorescence-activated cell sorting (FACS). In brief, freshly-isolated CF cells were pelleted down by centrifugation at 400 g for 5 minutes and resuspended in FACS buffer (PBS supplemented with 1% FCS and 1 mM EDTA). The cell suspensions were first blocked with anti-mouse CD16/CD32 (1:200; Clone 93, Thermo Fisher Scientific) for 10 min (4°C). The suspensions were then incubated on ice for 30 minutes with a combination of fluorochrome-conjugated antibodies (diluted in FACS buffer) against: the endothelial lineage marker CD31-PE (1:300, clone 390, Thermo Fisher Scientific), the VSMC/pericyte lineage marker PDGFR-β-PE (1:300, clone APB5, Novus Biologicals), the leukocyte lineage marker CD45-PE (1:300, clone 30-F11, Millipore), the mature fibroblast marker vimentin-APC [[11](#_ENREF_11)] (1:300, clone 280618, Novus Biologicals), and the mature fibroblast marker desmin-Alexa Fluor 647 [[11](#_ENREF_11)] (1:300, clone DES/1711, Novus Biologicals). DAPI (4’,6-diamidino-2-phenylindole, 1 µg/µl; Sigma-Aldrich) was used to distinguish live and dead cells. Mature CFs (defined as CD31^neg^PDGFR-β^neg^CD45^neg^ vimentin^pos^desmin^pos^ cells) were sorted by FACS with a BD Biosciences FACSAria III. Data were analyzed with FlowJo. Based on nine randomly-selected samples, FACS analysis revealed CF purity within the murine heart-derived CF cell fractions (mean ± standard deviation) to be 93.7 ± 3.7%.

**Isolation of neonatal rat cardiomyocytes and cardiac fibroblasts**

Neonatal rat CMs (NRCMs) and neonatal rat CFs (NRCFs) were prepared from Sprague-Dawley pups (aged 0-1 day) according to established procedure [[12](#_ENREF_12)]. In brief, pups were sacrificed by decapitation and hearts removed and dissociated by treatment at 37°C with pancreatin (Sigma-Aldrich) and type II collagenase in calcium and bicarbonate free Hank’s HEPES (CBFHH) buffer (20 mM HEPES, 0.5 mM KH_2_PO_4_, 0.3 mM Na_2_HPO_4_ buffered at pH 7.3 with 5 mM KCl, 120 mM NaCl, 0.8 mM MgSO_4_, and 5.6 mM glucose) supplemented with 1% pen-strep. At 10-minute intervals, dissociated cells were removed and added to a fresh tube of FCS. Enzymatic digest buffer was added anew to the remaining non-dissociated cardiac tissue for a further five rounds. The remaining adherent cells were primarily NRCFs. NRCF purification was performed with the mouse/rat CD90.1 MicroBead Kit using the MACS system (Miltenyi Biotec). The resulting CD90+ eluate (containing the purified NRCFs) was resuspended and cultured in MEM supplemented with 5% FCS and 1% pen-strep until 80-90% confluence prior to analysis.

The dissociated cells mentioned above were pooled and subjected to a centrifugation step (50 g, 5 minutes), followed by resuspension in MEM supplemented with 5% FCS, 40-μm filtration, and culturing in 100-mm Nunc dishes (Thermo Fisher Scientific) for 75 minutes (37°C, 1% CO_2_). At this point, the supernatant fraction containing the NRCMs was harvested. A cell counter (Countess Automated Cell Counter, Invitrogen, Thermo Fisher Scientific) enumerated viable NRCMs. The NRCMs were plated and cultured on MEM supplemented with 5% FCS and 1% pen-strep until 60-70% confluence prior to analysis.

**Evaluation of cardiomyocyte hypertrophy**

NRCM cells were seeded in MEM with FCS (1%) into glass-bottomed 96-well plates (Ibidi) suitable for high magnification optical imaging. NRCMs underwent transection with Lipofectamine 2000 (Invitrogen) 24 hour after plating with miR-27a-5p mimic or miR-Ctrl (50 nM; Thermo Fisher Scientific). The media was replaced 6 hours later with MEM/FCS (0.1%), and 48 hours after that with MEM/FCS (0.1%) with/without phenylephrine (PE, 50 µM; Sigma-Aldrich). Three days after NRCMs had been transfected, they were rinsed with PBS two times, underwent fixation (PFA, 4%, 10 minutes), permeabilization (0.2% Triton-X in PBS), and immunocytochemistry (ICC) staining within the 96-well culture plates according to established procedure [[12](#_ENREF_12)]. ICC was performed with a primary antibody against α-actinin (sarcomeric, 1:1,000, clone EA-53; Sigma-Aldrich) at 37°C for 45 minutes, followed by rinse steps, and a secondary antibody (Alexa Fluor 488-tagged, 1:200; Invitrogen) at 37°C for 30 minutes, and counterstained with DAPI. CMs were identified in the green channel (488 nm), while non-CMs were recognized by lack of α-actinin staining and were denoted in red.

**Quantitative real-time PCR (qPCR)**

A RNeasy Mini Kit (Qiagen) was employed to extract total RNA; SuperScript II Reverse Transcriptase (Invitrogen) was used to synthesize cDNA from a 500-ng aliquot of total RNA. FastStart Universal SYBR Green Master Mix (Roche, Basel, Switzerland) was used for qPCR in reactions (2 µl cDNA template, 400 nM primers, 1x SYBR Green Master Mix), which were analyzed on a StepOnePlus Real-Time PCR System (Applied Biosystems, Thermo Fisher Scientific). All the following primers targeted the murine genes: α-smooth muscle actin (*Acta2*) forward 5′-AGC AGA ACA GAG GAA TGC AGT GGA AGA GAC, reverse 5′-CCT CCC ACT CGC CTC CCA AAC AAG GAGC; collagen type I, alpha 1 (*Col1a1*) forward 5′-CTG GCA AGA AGG GAG ATG A, reverse 5′-CAC CAT CCA AAC CAC TGA AA; collagen type I, alpha 2 (*Col1a2*) forward 5′-AGG TCT TCC TGG AGC TGA TG, reverse 5′-ACC CAC AGG GCC TTC TTT AC; collagen type III, alpha 1 (*Col3a1*) forward 5′-ACA GCA AAT TCA CTT ACA CAG TTC, reverse 5′-CTC ATT GCC TTG CGT GTT T; fibronectin containing extra domain A (*Fn-EDA*) forward 5′-ATC GCC CTA AAG GAC TGG, reverse 5′-CAT CCT CAG GGC TCG AGT AG; total fibronectin (*Fn*) forward 5′-AAT GGA AAA GGG GAA TGG AC, reverse 5′-CTC GGT TGT CCT TCT TGC TC; housekeeping control glyceraldehyde 3-phosphate dehydrogenase (*Gapdh*) forward 5′-GTG AAG GTC GGT GTG AAC G, reverse 5′-TCG TTG ATG GCA ACA ATC TC; lysyl oxidase (*Lox*) forward 5′-ATG CCA ACA CAC AGA GGA GA, reverse 5′- AGG TGT CAT AAC ATC CAG GAC TC; myosin heavy chain 6 (*Myh6*) forward 5′-GCC CAG TAC CTC CGA AAG TC, reverse 5′-GCC TTA ACA TAC TCC TCC TTG TC; myosin heavy chain 7 (*Myh7*) forward 5′-ACT GTC AAC ACT AAG AGG GTC A, reverse 5′-TTG GAT GAT TTG ATC TTC CAG GG; atrial natriuretic peptide (*Nppa*) forward 5′-GCT TCC AGG CCA TAT TGG AG, reverse 5′-GGG GGC ATG ACC TCA TCT T; periostin (*Postn*) forward 5′-AAC CAA GGA CCT GAA ACA CG, reverse, 5′-CAA AGA GCG TGA AGT GAC CA; transforming growth factor-β1 (*Tgfb1*) forward 5′-CCC ACT CCC GTG GCT TC, reverse 5′-GTT CCA CAT GTT GCT CCA C; and transforming growth factor-β2 (*Tgfb2*) forward 5′-TGG AGT TCA GAC ACT CAA CACA, reverse 5′-AAG CTT CGG GAT TTA TGG TGT.

For miR-27a-5p qPCR evaluation, peqGOLD TriFast (Peqlab, VWR) was employed to purify total RNA from cardiac cells or tissue; a Universal cDNA Synthesis Kit II (Qiagen) was used to synthesize cDNA from a 10-ng aliquot of total RNA. FastStart Universal SYBR Green Master Mix (Roche) was used for qPCR reactions with primers for each miR-27a-5p (miRCURY LNA PCR primer) and for U6 standard (both Qiagen), which were then analyzed on a StepOnePlus Real-Time PCR System (Applied Biosystems). Primer efficiency were calculated by linear regression employing the Pfaffl method (LinRegPCR program).

**Western blotting**

Ventricular or cell lysates underwent sodium dodecyl sulfate polyacrylamide gel electrophoresis (SDS-PAGE) followed by transfer onto nitrocellulose membranes. Protein levels of loading control β-actin (1:1000; ab8227, Abcam), α-smooth muscle actin (α-SMA/Acta2, 1:1000; ab5694, Abcam), collagen type I (Col I, 1:1,000; ab34710, Abcam), collagen type III (Col III, 1:1000; ab7778, Abcam), Early Growth Response Protein 3 (Egr3, 1:1000; PA5-40841, Thermo Fisher Scientific), total fibronectin (Fn, 1:1,000; ab2413, Abcam), fibronectin containing extra domain A (Fn-EDA, 1:1000, MAB1940, Millipore), periostin (1:1,000, ab14041, Abcam), phospho-Smad2 (p-Smad2, 1:1000, #3108, Cell Signaling Technology), phospho-Smad3 (p-Smad3, 1:2000, ab52903, Abcam), total Smad2/3 (1:1000, #3102, Cell Signaling Technology), transforming growth factor-β1 (Tgf-β1, 1:1000, ab9758, Abcam), and transforming growth factor-β2 (Tgf-β2, 1:1000, ab113670, Abcam) were detected using the indicated primary antibodies. We then applied species-appropriate horseradish peroxidase (HRP)-conjugated secondary antibodies followed by 60 minutes of avidin-biotin peroxidase complex incubation (ABC HRP kit, Vector Laboratories). Blots were developed with enhanced chemiluminescence reagents (Life Technologies). Densitometry was performed by a blinded investigator using ImageJ. Western blot images were cropped to enable proper display in the figures. Full blot images are provided in Supp. Fig. 5.

**Analysis of the NRCF secretome**

NRCFs, isolated as described earlier, were plated to six-well cultures plates and underwent transfection the next day with antimiR-27a-5p LNA (50 nM), antimiR-Ctrl LNA (50 nM), miR-27a-5p mimic (50 nM), or miR-Ctrl (50 nM) using Lipofactamine 2000 (Invitrogen). The media was replaced 6 hours later with MEM/FCS (0.1%), and 48 hours after transfection, the conditioned media was harvested, and particulates were eliminated by centrifugation (3000g, 10 minutes). When a combination of siRNAs against rat Egr3 (NM_017086.1) (siEgr3.1, 5’-GCA ACA AGA CCG UGA CCUA-3’, 50 nM; siEgr3.2, 5’-GGU GCC AGG ACA ACA UCAU-3’, 50 nM) or control siRNA (siCtrl, 5’-GCA GAA CCA GTG CAA CCUA-3’, 100 nM) were used (Thermo Fisher Scientific), the siRNAs were added to the medium 6 h post-LNA transfection. The conditioned media supernatant was siphoned off to a fresh tube and kept at −80 °C until analysis.

Secretome analysis was performed on 2 ml of conditioned media, which was reduced to 100 μl volume by concentrating in spin column [3 kD molecular weight cut-off (MWCO)]. A 30 μl aliquot of Laemmli buffer (4×) was added and the proteins were resolved by gel electrophoresis (Bis-Tris, 4-12%, 130 V, 1.5 hours). A silver stain treatment was used to visualize protein bands within each lane. Next, each lane was excised and cut into 12 consecutive strips, which were individually treated with on a ProGest Protein Digestion Station (Digilab). The following day, sample preparation was performed by lyophilization and resuspension of the digested peptides in 20 μl of aqueous acetonitrile (ACN, 2%) and trifluoroacetic acid (TFA, 0.05%) solution. A 15 μl aliquot was then resolved by reverse phase nano-flow high-performance liquid chromatography (HPLC) using an Acclaim PepMap C18 column (25 cm × 75 μm) on an UltiMate 3000 RSLCnano System (both Thermo Fisher Scientific). The two mobile phases were: (i) solution A, formic acid (FA, 0.1%) in water, and (ii) solution B, ACN (80%), FA (0.1%) in water. The HPLC program was: interval 0-5 minutes, 2-10% B; interval 5-65 minutes, 10-30% B; interval 65-70 minutes, 30-40% B; interval 70-80 minutes, 99% B; interval 80-100 minutes, 2% B. Eluate was directly fed into an LTQ Orbitrap XL mass spectrometer (Thermo Fisher Scientific) operating in full ion scan mode, and in a mass-to-charge ratio (m/z) ranging from 350 to 1,600, resolution of 60,000 (m/z=400), and lock mass m/z of 445.12003. Tandem mass spectrometry (MS/MS) was implemented on the collision-induced dissociation (CID) from the top 6 ions using a dynamic exclusion of 120 seconds.

Mascot (version 2.3.01; Matrix Science) was used to compare the raw MS and MS/MS files to the UniProtKB/Swiss-Prot rat database (2014_01, which encompasses 7,894 protein entries). The following criteria were used: (i) the digest enzyme was trypsin, and two missing cleavages were permissible, (ii) mass tolerance limits were set to 10 ppm for the parent ions, and 0.8 Da for the fragmented ions, and (iii) fixed modifications used cysteine carboxyamidomethylation while variable modifications used proline, lysine, and methionine oxidation. Scaffold (version 4.3.2; Proteome Software) identified proteins according to the following criteria: (i) peptides probability > 95%, (ii) protein probability > 99%, and (iii) at least two unique peptides identified. Standardized spectrum counts were employed for additional analysis.

***In silico* Venn analysis**

The TargetScan database (www.targetscan.org) was interrogated for putative targets of human hsa-miR-27a-5p, mouse mmu-miR-27a-5p, and rat rno-miR-27a-5p. Only putative targets possessing a cumulative weighted context++ score of less than -0.40 were selected for inclusion in the Venn analysis. In addition, the GEO dataset GSE18224 [[13](#_ENREF_13)] was interrogated for genes that were upregulated in the left ventricular transcriptome of WT TAC mice (n=8 mice) relative to WT sham mice (n=8 mice). Only upregulated genes possessing an absolute fold-change of greater than 1.50 and an adjusted *p*-value of less than 0.05 were selected for inclusion in the Venn analysis. Putative target genes in the overlap of all four sets were deemed potential targets of miR-27a-5p that are upregulated by TAC.

**Luminescence reporter assays**

A luminescence reporter assay of Tgf-β activity was performed with a plasmid with a WT SBE binding sequence (TOPflash plasmid; Addgene catalog no. 12456) and a respective control with a mutant SBE binding sequence (FOPflash plasmid; Addgene catalog no. 12457) [[14](#_ENREF_14)]. NRCFs underwent transfection with Lipofactamine 2000 (Invitrogen) of SBE reporter with one of the following: antimiR-27a-5p LNA (50 nM), antimiR-Ctrl LNA (50 nM), miR-27a-5p mimic (50 nM), or miR-Ctrl (50 nM). Tgf-β (50 ng/ml; Cell Guidance Systems, Cambridge UK) was added 6 hours prior to the determination of luminescence signal with a One-Glo Luciferase assay System (Promega).

The base pairs spanning the 3′-untranslated region (3′-UTR) of human EGR3, which includes the two putative conserved miR-27a-5p binding sequences, were amplified and ligated into a dual-fluorophore vector. The first fluorophore, eGFP (enhanced GFP), was located downstream of the EGR3 3′-UTR and its signal intensity quantified miR-27a-5p binding. The second fluorophore, tdTomato, served as a standard of transfection efficiency. A Q5 Site-Directed Mutagenesis Kit (New England Biolabs) was used to mutate the miR-27a-5p binding sequence. HEK293 cells in optical-grade 96-well culture plates (Ibidi) in MEM/FCS (5%) underwent transfection with Lipofactamine 2000 (Invitrogen) of: (i) EGR3 3′-UTR dual-fluorophore plasmids or mutant controls, and (ii) miR-27a-5p mimic (50 nM) or miR-Ctrl (50 nM).

**Immunofluorescence**

NRCFs were treated for immunofluorescence as previously described [[15](#_ENREF_15)]: fixation under 3% (v/v) paraformaldehyde in PBS for 20 minutes, formaldehyde quenching with 0.2 M glycine in PBS for 20 minutes, permeabilization with 0.5% (v/v) Triton X (Sigma-Aldrich) in PBS for 4 minutes (20°C), blocking with 3% (w/v) bovine serum albumin (BSA) for 60 minutes, primary incubation with anti-rat Ltbp1 primary antibody (1:50, ab78294, Abcam) diluted in 3% (w/v) BSA for 60 minutes, secondary incubation with fluorescent secondary antibody (Jackson ImmunoResearch Laboratories) diluted in 3% (w/v) BSA for 45 minutes in the darkkroom, and nuclear counterstaining with DAPI (300 nM; Sigma-Aldrich) for 5 minutes in the darkroom. Coverslips were mounted with Prolong Antifade Mounting Medium (Molecular Probes), sealed with nail varnish, and briefly stored in the darkroom (4°C). Slides were examined and captured with a Leica TCS SP5 II confocal microscope (Leica Microsystems).

**Statistical analyses**

We employed GraphPad Prism version 6 to perform all statistical tests. All data was expressed as means ± standard errors of the mean (SEMs). Normality of data distribution was confirmed using Kolmogorov-Smirnov (K-S) or Shapiro-Wilk tests. Common variance was evaluated using Bartlett’s method or F-test. Comparisons between two groups was evaluated by Student’s *t*-test; comparisons between multiple groups was evaluated by one- or two-way analysis of variance (ANOVA) with post-hoc Bonferroni testing where appropriate. Results were statistically significant at a *P*-value of less than 0.05.

**SUPPLEMENTARY FIGURE LEGENDS**

**Supplementary Figure 1. miR-27a-5p does not stimulate cardiomyocyte hypertrophy *in vitro***

**(A)** Effect of the miR-27a-5p on luciferase reporter activity in primary neonatal rat cardiomyocytes (NRCMs). The luciferase reporter contains a miR-27a-5p binding sequence downstream to the luciferase gene; NRCMs underwent co-transfection with the miR-27a-5p luciferase reporter and either miR-27a-5p mimic (50 nM) or miR-Ctrl (50 nM). n=3 biological replicates × 3 technical replicates. **P*<0.05, ***P*<0.01 vs. miR-Ctrl [one-way ANOVA with post-hoc Bonferroni]. **(B)** NRCMs underwent transfection with miR-27a-5p mimic or miR-Ctrl, incubated with 50 μM phenylephrine (PE; a α1-adrenergic receptor agonist and promoter of hypertrophy) for 48 hours, followed by fixation and staining with an anti-α-actinin antibody and DAPI. NRCM identification and NRCM size determination were performed by a software program, which scored NRCM size (green) and identified non-CM contaminants (α-actinin-negative, depicted in red); scale bar = 100 μm. n=3 biological replicates × 3 technical replicates. **P<*0.05, ***P<*0.01 vs. matching –PE group; †*P*<0.05, ††*P*<0.01 vs. matching miR-Ctrl group [two-way ANOVA with post-hoc Bonferroni]. Data expressed as means ± standard errors of the mean (SEMs).

**Supplementary Figure 2. Body mass and lung, liver, and kidney fibrosis assessments in wild-type and miR-27a−/− mice**

**(A)** Body mass comparisons between untreated wild-type (WT) and miR-27a−/− mice (male, aged 3 months). n = 9 animals per cohort. Comparisons performed by one-way ANOVA with post-hoc Bonferroni. **(B)** Typical Picrosirius Red/Fast Green FCF-stained lung, liver, and kidney tissue sections in WT and miR-27a−/− (KO) mice 21 days after the transverse aortic constriction (TAC) procedure; scale bar = 100 µm. Data expressed as means ± standard errors of the mean (SEMs).

**Supplementary Figure 3. Assessment of hypertrophy biomarker expression in the transverse aortic constriction murine models**

Assessment of atrial natriuretic peptide (*Nppa*) mRNA expression and myosin heavy chain 7/myosin heavy chain 6 (*Myh7*/*Myh6*) mRNA ratio in left ventricle tissue by quantitative real-time PCR (qPCR) in transverse aortic constriction (TAC)-treated **(A)** miR-27a−/− and wild-type (WT) animals; **(B)** anti-miR-27a-5p LNA (AntimiR-27a-5p), control LNA (AntimiR-Ctrl), and vehicle (PBS) animals; **(C)** AAV2-Postn-iCre and AAV2-Ctrl animals; and **(D)** AAV2-Postn-miR-27a and AAV2-Ctrl animals. Comparisons performed by Student’s *t*-test or one-way ANOVA with post-hoc Bonferroni. Data expressed as means ± standard errors of the mean (SEMs).

### Supplementary Figure 4. Cardiac fibroblast miR-27a-5p levels decline with age and transverse aortic constriction-induced stress

Assessment of cardiac fibroblasts (CF) miR-27a-5p levels by quantitative real-time PCR (qPCR). **(A)** Left ventricular tissue-derived CF miR-27a-5p levels over time in untreated wild-type (WT) mice; n = 9 animals per cohort. **P<*0.05, ***P<*0.01 vs. Week 1; †*P<*0.05, ††*P<*0.01 vs. Week 3; ‡*P<*0.05, ‡‡*P<*0.01 vs. Week 8 [one-way ANOVA with post-hoc Bonferroni]. **(B)** Left ventricular tissue-derived CF miR-27a-5p levels in sham- or transverse aortic constriction (TAC)-operated animals; n = 9 animals per cohort. **P<*0.05, ***P<*0.01 vs. Sham [one-way ANOVA with post-hoc Bonferroni]. **(C, D)** miR-27a-5p levels in **(C)** primary neonatal rat cardiac fibroblasts (NRCFs) and **(D)** primary adult mouse cardiac fibroblasts (AMCFs) fresh in culture and after two weeks in continuous culture. n=3 biological replicates × 3 technical replicates. **P<*0.05, ***P<*0.01 vs. Fresh [Student’s *t*-test]. Data expressed as means ± standard errors of the mean (SEMs).

**Supplementary Figure 1**


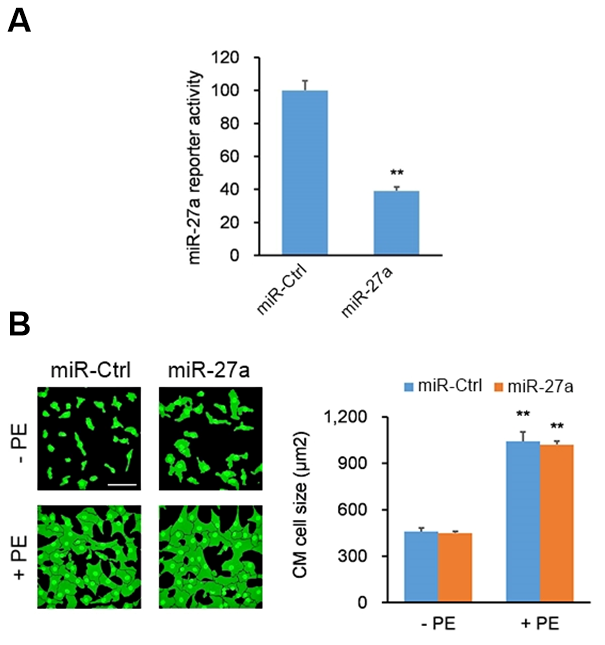


**Supplementary Figure 2**

**
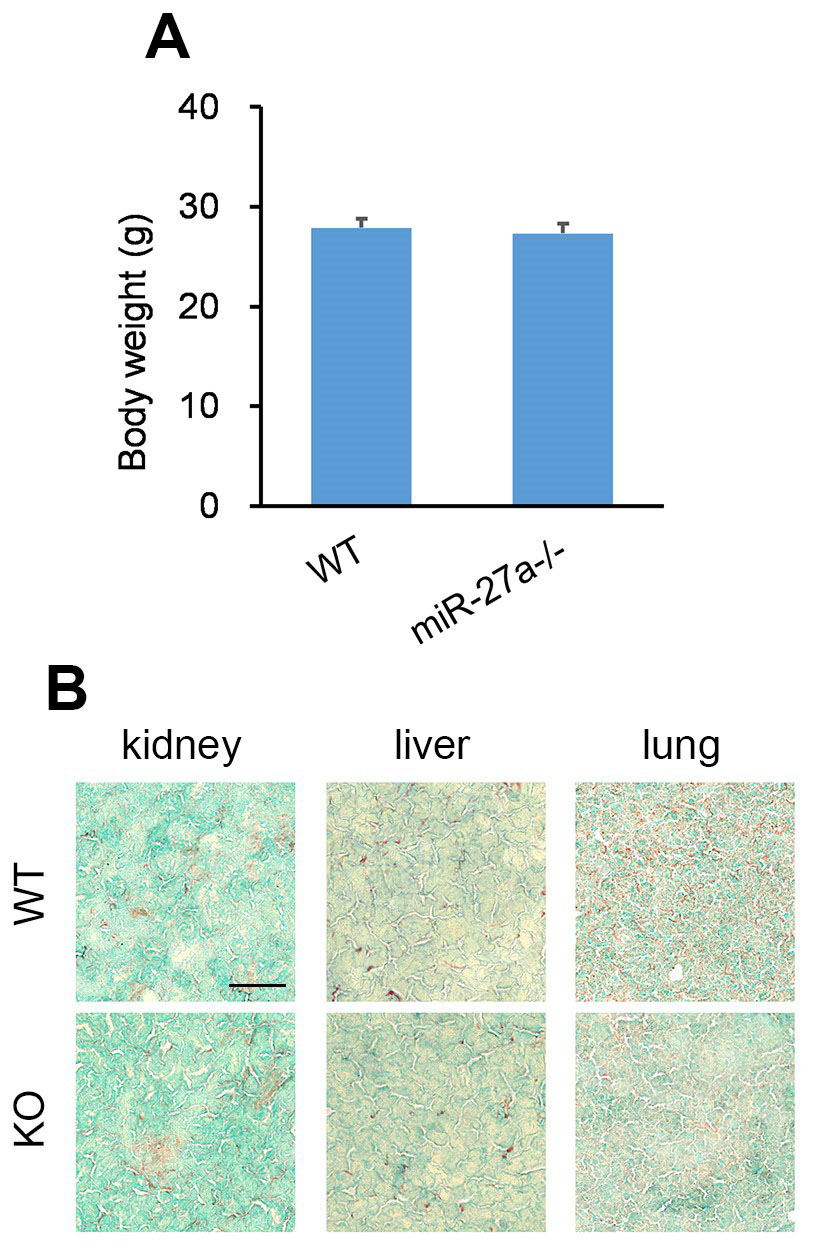
**

**Supplementary Figure 3**


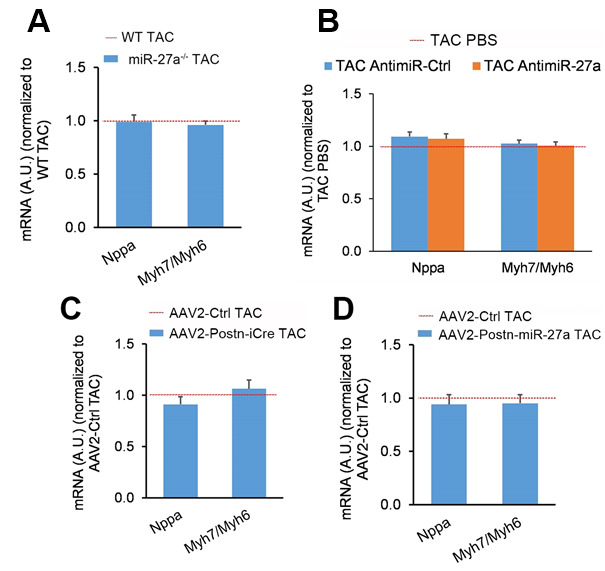


**Supplementary Figure 4**


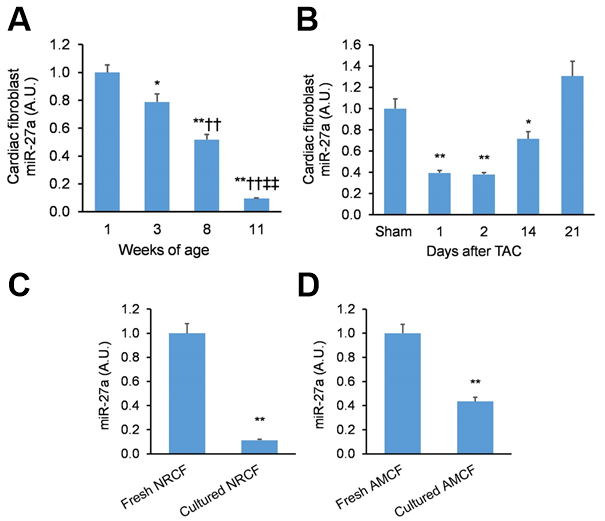


**Supplementary Figure 5**

**Figure 1G**

**
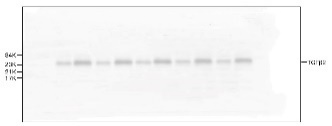

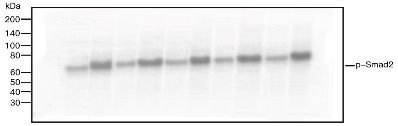
**Tg
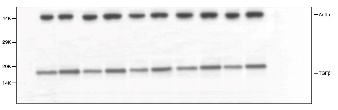
fβ1 Tgfβ2 p-Smad2

p-Smad**
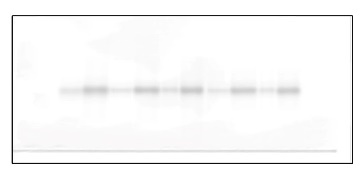

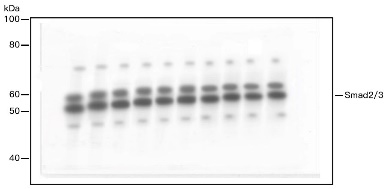

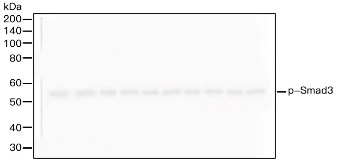
**3 Smad2/3 CoI I

**
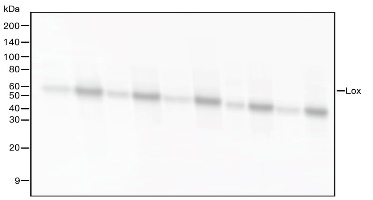
**CoI III Lox

**
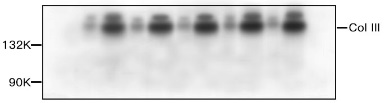
**

**Figure 1I**

α-
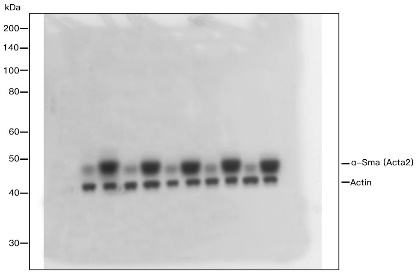
Sma (Acta2) Fn-EDA Total Fn


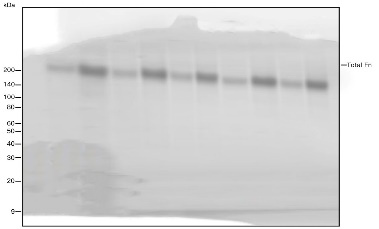

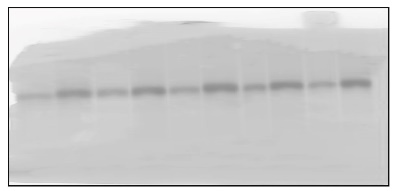


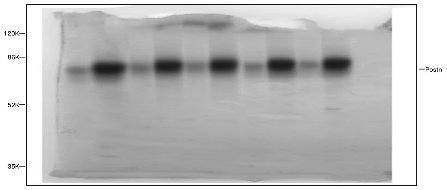
Postn

**Figure 2H**


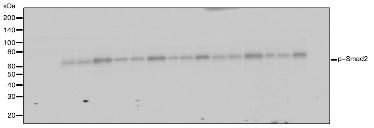

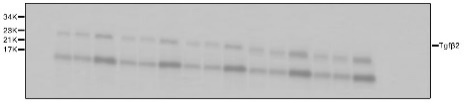
T
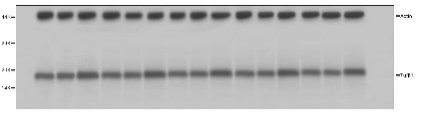
gfβ1 Tgfβ2 p-Smad2


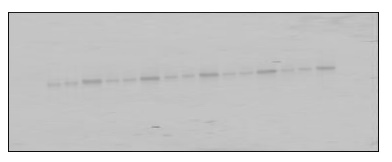

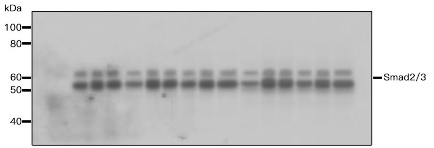
p-Smad3 Smad2/3 CoI I


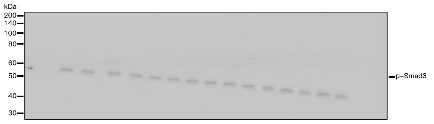


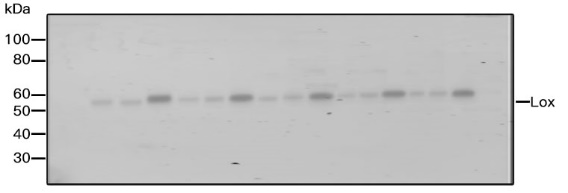

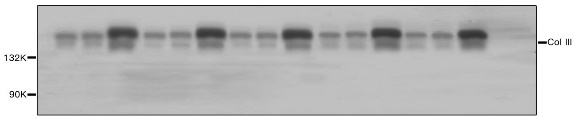
CoI III Lox

**Figure 2J**


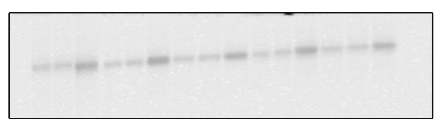

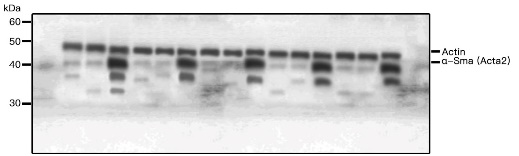
α-Sma (Acta2) Fn-EDA Total Fn


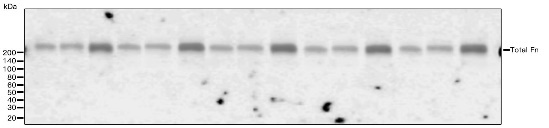


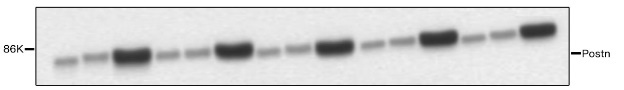
Postn

**Figure 3H**


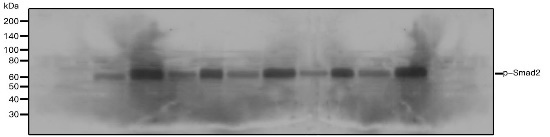

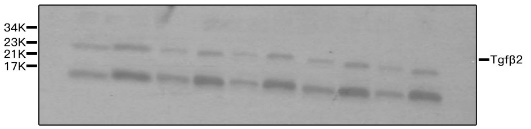
Tgfβ1 Tgfβ2 p-Smad2


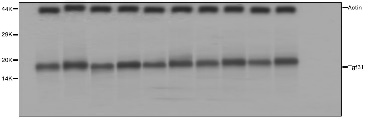


p-Smad3 Smad2/3 CoI I


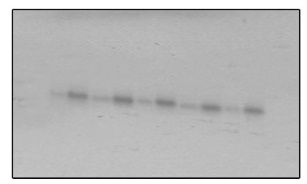

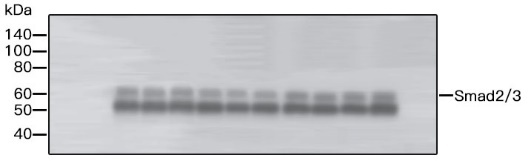

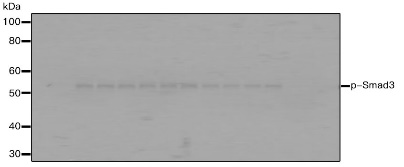


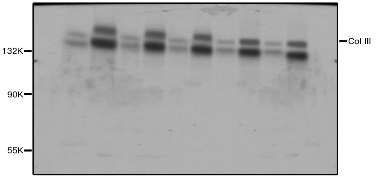
CoI III Lox


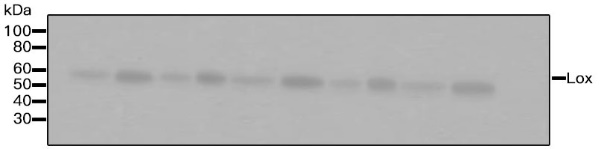


**Figure 3J**


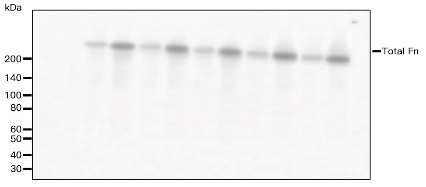

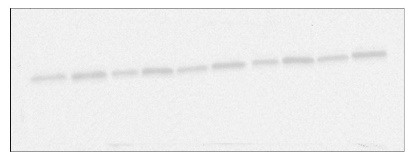
α-Sma
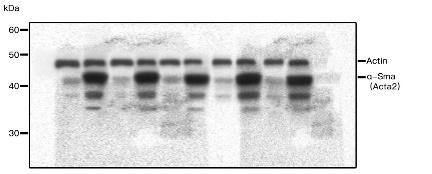
 (Acta2) Fn-EDA Total Fn


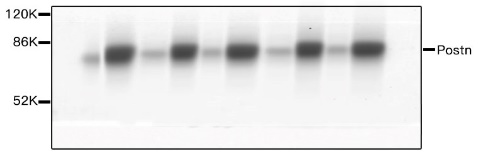
Postn

**Figure 4I**


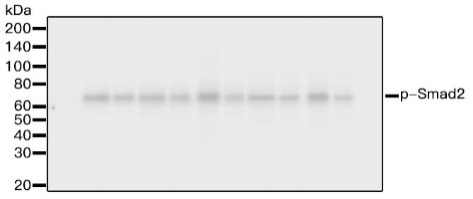

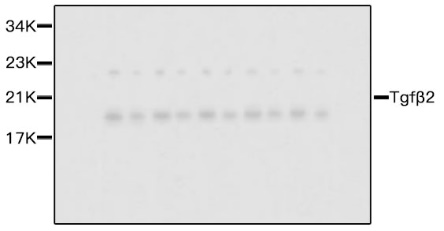
Tgfβ1 Tgfβ2 p-Smad2


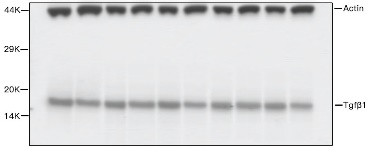


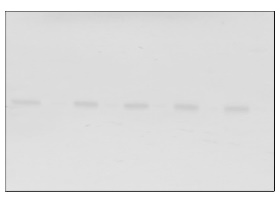

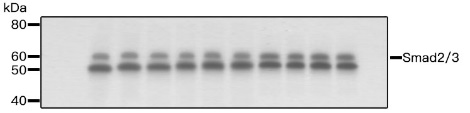

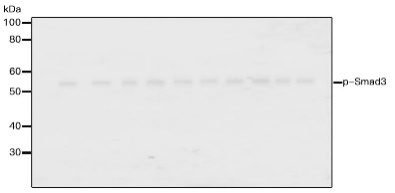
p-Smad3 Smad2/3 CoI I

CoI III Lox


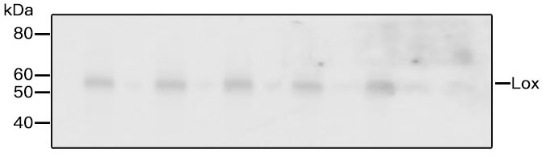

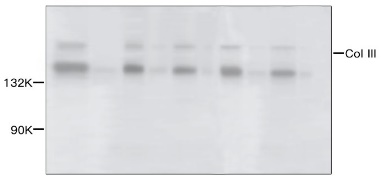


**Figure 4K**


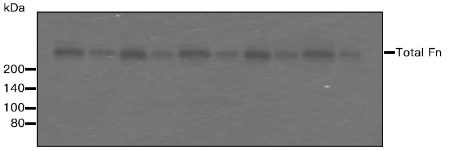

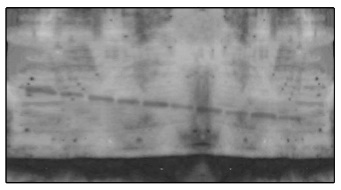
α-Sma
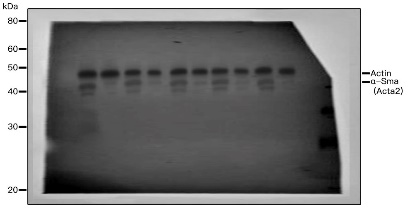
 (Acta2) Fn-EDA Total Fn

Postn


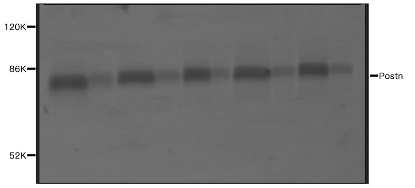


**Figure 5I**


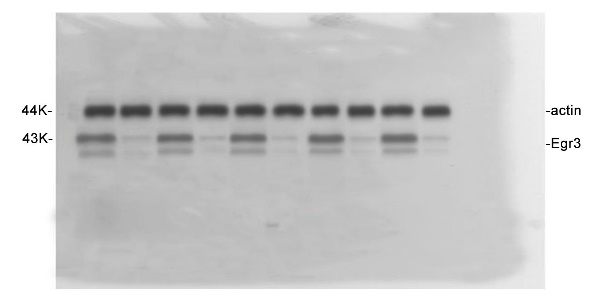


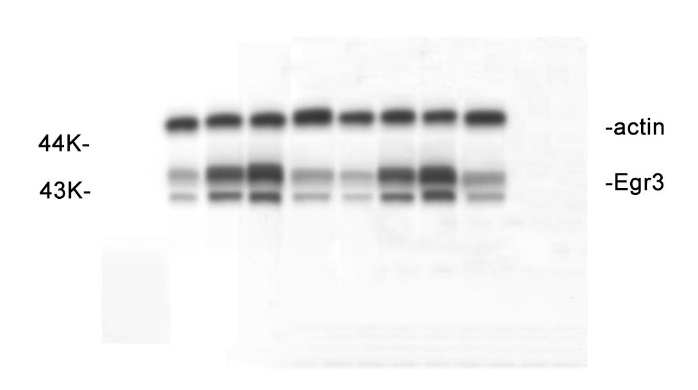


**Figure 5J**


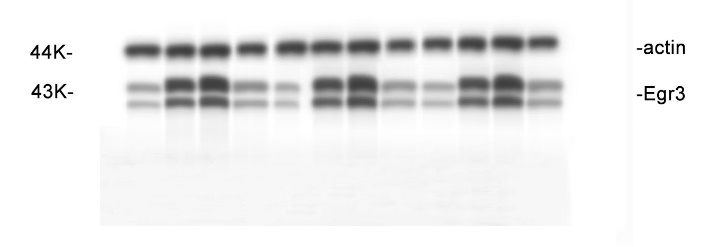


**REFERENCES FOR SUPPLEMENTARY INFORMATION**

1. **Liu F, Chen J, Wang P, Li H, Zhou Y, Liu H, Liu Z, Zheng R, Wang L, Yang H.** MicroRNA-27a controls the intracellular survival of Mycobacterium tuberculosis by regulating calcium-associated autophagy. *Nature communications*. 2018; 9: 4295.

2. **Remenyi J, van den Bosch MW, Palygin O, Mistry RB, McKenzie C, Macdonald A, Hutvagner G, Arthur JSC, Frenguelli BG, Pankratov Y.** miR-132/212 knockout mice reveal roles for these miRNAs in regulating cortical synaptic transmission and plasticity. *PloS one*. 2013; 8: e62509.

3. **Li M, Jayandharan GR, Li B, Ling C, Ma W, Srivastava A, Zhong L.** High-efficiency transduction of fibroblasts and mesenchymal stem cells by tyrosine-mutant AAV2 vectors for their potential use in cellular therapy. *Human gene therapy*. 2010; 21: 1527-43.

4. **Takeda N, Manabe I, Uchino Y, Eguchi K, Matsumoto S, Nishimura S, Shindo T, Sano M, Otsu K, Snider P.** Cardiac fibroblasts are essential for the adaptive response of the murine heart to pressure overload. *The Journal of clinical investigation*. 2010; 120: 254-65.

5. **Sassi Y, Avramopoulos P, Ramanujam D, Grüter L, Werfel S, Giosele S, Brunner A-D, Esfandyari D, Papadopoulou AS, De Strooper B.** Cardiac myocyte miR-29 promotes pathological remodeling of the heart by activating Wnt signaling. *Nature communications*. 2017; 8: 1614.

6. **Ramanujam D, Sassi Y, Laggerbauer B, Engelhardt S.** Viral vector-based targeting of miR-21 in cardiac nonmyocyte cells reduces pathologic remodeling of the heart. *Molecular Therapy*. 2016; 24: 1939-48.

7. **Rockman HA, Ross RS, Harris AN, Knowlton KU, Steinhelper ME, Field LJ, Ross J, Chien KR.** Segregation of atrial-specific and inducible expression of an atrial natriuretic factor transgene in an in vivo murine model of cardiac hypertrophy. *Proceedings of the National Academy of Sciences*. 1991; 88: 8277-81.

8. **Moore JB, Tang X-L, Zhao J, Fischer AG, Wu W-J, Uchida S, Gumpert AM, Stowers H, Wysoczynski M, Bolli R.** Epigenetically modified cardiac mesenchymal stromal cells limit myocardial fibrosis and promote functional recovery in a model of chronic ischemic cardiomyopathy. *Basic research in cardiology*. 2019; 114: 3.

9. **Viereck J, Kumarswamy R, Foinquinos A, Xiao K, Avramopoulos P, Kunz M, Dittrich M, Maetzig T, Zimmer K, Remke J.** Long noncoding RNA Chast promotes cardiac remodeling. *Science translational medicine*. 2016; 8: 326ra22-ra22.

10. **Weber SC, Gratopp A, Akanbi S, Rheinlaender C, Sallmon H, Barikbin P, Koehne PS.** Isolation and culture of fibroblasts, vascular smooth muscle, and endothelial cells from the fetal rat ductus arteriosus. *Pediatric research*. 2011; 70: 236-41.

11. **Hur W, Lee HY, Min HS, Wufuer M, Lee C-w, Hur JA, Kim SH, Kim BK, Choi TH.** Regeneration of full-thickness skin defects by differentiated adipose-derived stem cells into fibroblast-like cells by fibroblast-conditioned medium. *Stem cell research & therapy*. 2017; 8: 92.

12. **Jentzsch C, Leierseder S, Loyer X, Flohrschütz I, Sassi Y, Hartmann D, Thum T, Laggerbauer B, Engelhardt S.** A phenotypic screen to identify hypertrophy-modulating microRNAs in primary cardiomyocytes. *Journal of molecular and cellular cardiology*. 2012; 52: 13-20.

13. **Fliegner D, Schubert C, Penkalla A, Witt H, Kararigas G, Dworatzek E, Staub E, Martus P, Noppinger PR, Kintscher U.** Female sex and estrogen receptor-β attenuate cardiac remodeling and apoptosis in pressure overload. *American Journal of Physiology-Regulatory, Integrative and Comparative Physiology*. 2010; 298: R1597-R606.

14. **Veeman MT, Slusarski DC, Kaykas A, Louie SH, Moon RT.** Zebrafish prickle, a modulator of noncanonical Wnt/Fz signaling, regulates gastrulation movements. *Current Biology*. 2003; 13: 680-5.

15. **Kinsey R, Williamson MR, Chaudhry S, Mellody KT, McGovern A, Takahashi S, Shuttleworth CA, Kielty CM.** Fibrillin-1 microfibril deposition is dependent on fibronectin assembly. *Journal of cell science*. 2008; 121: 2696-704.
